# Intracellular marriage of bicarbonate and Mn ions as “immune ion reactors” to regulate redox homeostasis and enhanced antitumor immune responses

**DOI:** 10.1186/s12951-022-01404-x

**Published:** 2022-04-19

**Authors:** Yushuo Feng, Yaqing Liu, Xiaoqian Ma, Lihua Xu, Dandan Ding, Lei Chen, Zongzhang Wang, Ruixue Qin, Wenjing Sun, Hongmin Chen

**Affiliations:** State Key Laboratory of Molecular Vaccinology and Molecular Diagnostics and Center for Molecular Imaging and Translational Medicine, School of Public Health, Xiamen University, Xiamen, 361102 China

**Keywords:** Self-supplying intracellular ions, Redox homeostasis, Immune activator, Manganese immunotherapy, Orthotopic liver cancer

## Abstract

**Background:**

Different from Fe ions in Fenton reaction, Mn ions can function both as catalyst for chemodynamic therapy and immune adjuvant for antitumor immune responses. In Mn-mediated Fenton-like reaction, bicarbonate (${\text{HCO}}_{3}^{ - }$), as the most important component to amplify therapeutic effects, must be present, however, intracellular ${\text{HCO}}_{3}^{ - }$ is strictly limited because of the tight control by live cells.

**Results:**

Herein, Stimuli-responsive manganese carbonate-indocyanine green complexes (MnCO_3_-ICG) were designed for intracellular marriage of bicarbonate and Mn ions as “immune ion reactors” to regulate intracellular redox homeostasis and antitumor immune responses. Under the tumor acidic environment, the biodegradable complex can release “ion reactors” of Mn^2+^ and ${\text{HCO}}_{3}^{ - }$, and ICG in the cytoplasm. The suddenly increased ${\text{HCO}}_{3}^{ - }$ in situ inside the cells regulate intracellular pH, and accelerate the generation of hydroxyl radicals for the oxidative stress damage of tumors cells because ${\text{HCO}}_{3}^{ - }$ play a critical role to catalyze Mn-mediated Fenton-like reaction. Investigations in vitro and in vivo prove that the both CDT and phototherapy combined with Mn^2+^-enhanced immunotherapy effectively suppress tumor growth and realize complete tumor elimination.

**Conclusions:**

The combination therapy strategy with the help of novel immune adjuvants would produce an enhanced immune response, and be used for the treatment of deep tumors in situ.

**Supplementary Information:**

The online version contains supplementary material available at 10.1186/s12951-022-01404-x.

## Background

Chemodynamic therapy (CDT) is a potential therapeutic strategy due to its noninvasive nature and high selectivity [1, 2]. CDT is defined as in situ treatment via a Fenton/Fenton-like reaction (Fe^2+^/Mn^2+^) to generate reactive oxygen species (ROS) from the decomposition of endogenous hydrogen peroxide (H_2_O_2_) [3–7]. This process leads to oxidative stress from biomolecular substances such as proteins, lipids, and nucleic acids in tumor cells that are destroyed to induce cell death [8–17].

As an essential nutritional inorganic trace dietary element, manganese (Mn) is required for various physiological processes. Laboratory-synthesized Mn-based formulations (mainly MnO_x_) can deplete GSH biosynthesis, regulate pH, and decompose the H_2_O_2_ [18–22]. Unlike the Fe-based Fenton reaction, the Mn-mediated Fenton-like reaction requires bicarbonate (${\text{HCO}}_{3}^{ - }$), which is the most important component to amplify therapeutic effects [23]. The development of stimuli-responsive nanomedicine is appealing to achieve effective Mn-mediated Fenton-like cancer treatment. Thus, as a typical pH-dissociable Mn-based biomineral, MnCO_3_ formulations have excellent potential to achieve these purposes because they have similar biodegradability and biocompatibility as CaCO_3_ [24–27].

The enhancement of tumor therapy relies not only on the development of biocompatible nanoformulations but also on the discovery and implementation of clinically capable therapeutic mechanisms. Over the past decades, manganese-based nanostructures have drawn much attention in biomedical applications. For instance, Mn^2+^ salts or complexes have been used in clinical MR imaging [28–31] and can induce tumor cell apoptosis [15–17, 22, 27, 32]. Recent studies have revealed that Mn can lead to excellent tumor-ablation therapeutic effect, although the mechanism and role of Mn have not been completely explored. More evidences have indicated that there are two explanations for the enhanced therapy efficacy:

(1) a Mn-mediated Fenton-like reaction is strongly enhanced by ${\text{HCO}}_{3}^{ - }$.[33–37] Carbonate concentration and pH play essential roles in the formation of catalytically active species that maintains the high oxidation reactivity of the oxidizing intermediate [38, 39]. Mammalian tissues are bathed in a milieu that typically contains ${\text{HCO}}_{3}^{ - }$ at a concentration of about 25 mM, and cells have developed a mechanism to maintain an intracellular concentration of about 12 mM [40]. CO_2_ is the end product of mitochondrial energy production and can ultimately be converted into ${\text{HCO}}_{3}^{ - }$ in the cytoplasm [40–42]. In an acidic environment, carbonate reacts with the acid to quickly generate CO_2_ bubbles [43, 44], which regulate the local intracellular pH and generate ${\text{HCO}}_{3}^{ - }$ in situ; this boosts the Mn-mediated Fenton-like reaction. Governing pH regulation using bicarbonate has been carefully studied, and CO_2_/${\text{HCO}}_{3}^{ - }$ is important for maintaining uniform alkaline pH in small, non-vascularized tumors; it is an important target for cancer progression [45, 46]. The Gillies group comprehensively showed that biocarbonate plays an indispensable role in cancer management, including inhibiting spontaneous metastases and improving antitumor responses to immunotherapy [47]. A common formula, sodium bicarbonate, known as baking soda, is widely used in the clinic as an antiacid for treating gastric hyperacidity [48]. The Hu group completed a clinical trial into targeting intratumoral lactic acidosis-transarterial chemoembolization for large hepatocellular carcinoma (HCC) (TILA-TACE) (Clinical trial number: ChiCTR-IOR-14005319) [49]. Following the TILA-TACE procedure (widely employed for the local control of HCC), sodium bicarbonate plus cytotoxic drugs resulted in a high local control rate [50]. The authors stated that the possible reason is related to acidosis in the tumor microenvironment. Although this is a small-scale pilot study with limitations on cancer types and delivery method (sodium bicarbonate highly hydrolyzed), the results still offered the potential to exploit tumor acidosis from “the passenger” to “the driver’s seat” [40, 48, 51, 52]. This was done in combination with other anticancer therapies in treating various malignancies.

(2) Mn^2+^ strongly promotes immune responses. Immunotherapy has revolutionized clinical cancer treatment and reached conspicuous successes. Recent studies suggest that Mn^2+^ would strongly promote immune responses through proliferating cytotoxic T lymphocyte and promoting dendritic cell maturation [17, 53–55]. These reports imply that the development of Mn-based complexes combined with traditional treatment of cancer have great potential to improve the antitumor effect [15, 56]. Currently, only six adjuvants have been approved for clinical use, including Alum, MF59, AS04, AS03, AS01 and CpG 1018 [57]. Thus, developing novel adjuvants could induce a high magnitude of immunity response. Nanosized sustained release system can enhance the magnitude, quality, and persistence of immunity responses.

**Scheme 1:**
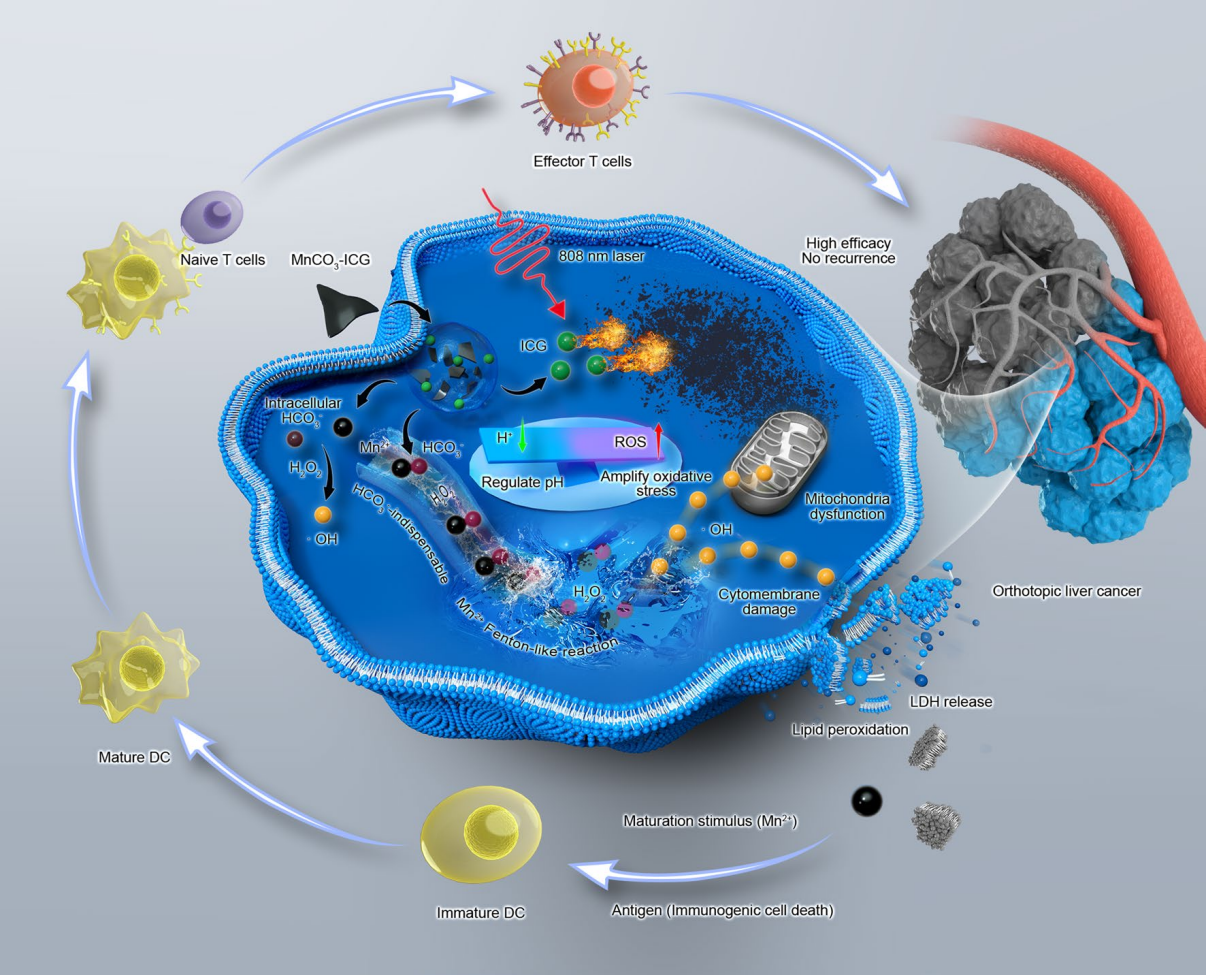
Schematic representation shows that intracellular marriage of bicarbonate and Mn ions as “immune ion reactors” to regulate redox homeostasis and antitumor immune responses for synchronous enduring orthotopic cancer treatment. In the acidic environment, MnCO_3_-ICG are decomposed to release ions (Mn^2+^, ${\text{HCO}}_{3}^{ - }$) in situ that regulates intracellular redox homeostasis and antitumor immune responses. The self-supplying ${\text{HCO}}_{3}^{ - }$ escalates tumor oxidative stresses by catalyzing Mn-based Fenton-like reaction to efficiently generate hydroxyl radicals (·OH); the released ICG amplifies tumor oxidative stress by photothermal process; and Mn^2+^ ions as one of adjuvants enhance the immune response

Herein, on the basis of the insights from the preliminary results, we designed a MnCO_3_-based nanocomplexes with amplification of tumor oxidative stress, regulation of intracellular redox homeostasis, and enhancement of immune response (Scheme 1). In our design, indocyanine green (ICG), an FDA-approved clinically used fluorescent agent for diagnostics and imaging-guided surgery, first chelates with Mn ions and co-condenses with CO_2_ to produce Mn carbonate-indocyanine green complexes (MnCO_3_-ICG) [58, 59]. The complexation of ICG and Mn ions facilitates efficient loading of imaging agents and also minimizes the susceptibility of Mn ions to intrahost oxidative stress before their intracellular release. Meanwhile, the MnCO_3_ is sacrificed via hydrolysis and releasing Mn^2+^ ions, ICG, and ${\text{CO}}_{3}^{2 - }$ into the cytosol. Compared with the other Mn-induced CDT strategies by solely increasing the intracellular Mn accumulation, the in situ self-supplied ${\text{HCO}}_{3}^{ - }$-pool serves as “immune ion reactors” to increase the ROS generation in the tumor cells and boost DC maturation and increase CD8^+^ T cells. The released ICG molecules could penetrate into deep tumors and further increase the therapeutic effects by imaging-guided phototherapy. Considering the negligible systemic biological toxicity of Mn^2+^, ${\text{CO}}_{3}^{2 - }$, and ICG, we believe the released components from the pH-triggered decomposition of MnCO_3_-ICG formulation is anticipated to act synergistically to amplify the oxidative stress, regulate intracellular redox homeostasis and activate the immune response, leading to enhanced tumor cell death in an on-demand manner.

## Materials and methods

### Synthesis of manganese carbonate-indocyanine green complexes (MnCO_3_-ICG)

MnCO_3_-ICG complexes were synthesized by adopting the gas diffusion process. Briefly, MnCl_2_·4H_2_O (10 mg/mL, 5 mL) and different concentrations of ICG were mixed with 50 mL ethanol. After stirred 3 h, the mixture (Mn-ICG) was placed in a sealed container with NH_4_HCO_3_ and kept at room temperature. After 0.5, 1, 2 or 4 h, the green MnCO_3_-ICG complexes were collected and purified by repeated centrifugation at 5000 rpm. The PAH (5 mg/mL) solution was added into 50 mL of ethanol containing different concentrations of MnCO_3_-ICG complexes to obtain excellent aqueous solubility. The mixture was then stirred for 1 h at room temperature. Finally, the obtained MnCO_3_-ICG complexes were collected and purified by centrifugation at 10,000 rpm for 10 min.

### The hydroxyl radicals (·OH) generation by Mn^2+^-mediated Fenton-like reaction

MnCO_3_-ICG ([Mn]: 50 μg/mL) were incubated with different buffer solution (pH 5.8; pH 7.4 with 20 mM NaHCO_3_; pH 7.4 with 20 mM NaHCO_3_) for 30 min. After centrifugation, the MB (10 μg/mL) and H_2_O_2_ (1 mM) were added to the supernatant. The absorbance of MB was measured after shaking at 37 °C for 30 min.

### Enhanced-chemodynamic activity of MnCO_3_-ICG complexes

MnCO_3_-ICG ([Mn]: 50 μg/mL) were incubated with different different concentrations of NaHCO_3_ in the acidic environment for 30 min. After centrifugation, the MB (10 μg/mL) and H_2_O_2_ (1 mM) were added to the supernatant. The absorbance of MB was measured after shaking at 37 °C for 30 min.

MnCO_3_-ICG and Mn-ICG ([Mn]: 50 μg/mL) were incubated with different buffer solution (pH 7.4; pH 5.8; pH 7.4 with 1 mM H_2_O_2_; pH 5.8 with 1 mM H_2_O_2_) for 30 min. After centrifugation, the MB (10 μg/mL) and H_2_O_2_ (1 mM) were added to the supernatant. The absorbance of MB was measured after shaking at 37 °C for 30 min.

MnCO_3_-ICG ([Mn]: 50 μg/mL) were incubated in the acidic environment (pH 5.8) for different time. After centrifugation, the MB (10 μg/mL) and H_2_O_2_ (1 mM) were added to the supernatant. The absorbance of MB was measured after shaking at 37 °C for 30 min.

### Cell uptake

To evaluate the cell uptake efficiency, 4T1 cells were seeded in observation dish at a density 10^5^ cells for 24 h. After that, the cells incubated with MnCO_3_-ICG ([Mn]: 5 μg/mL) in the dark for different time (2, 4, 8, 12, and 24 h). After washing with PBS for three times, the cells were stained with hoechst33342 (5 μg/mL) and imaged by a laser scanning confocal fluorescence microscope.

For quantitative analysis the cell uptake efficiency, 4T1 cells were seeded in six-well plates at a density 10^5^ cells for 24 h. After that, the cells incubated with MnCO_3_-ICG ([Mn]: 5 μg/mL) in the dark for different time (2, 4, 8, 12, and 24 h). After washing with PBS for three times, the cells were digested and analyzed by flow cytometer (ACEA Biosciences).

4T1 cells were also seeded in observation dish at a density 10^5^ cells for 24 h, and incubated with MnCO_3_-ICG ([Mn]: 5 μg/mL) in the dark for 24 h. After washing with PBS for three times, the cells were stained with hoechst33342 (5 μg/mL) and lyso-tracker (2 μM). The stained cells were imaged by a laser scanning confocal fluorescence microscope.

### In vitro photothermal and chemodynamic therapy effect

To evaluate the chemodynamic therapy effect, 4T1 cells, U87MG cells and Hep G2 cells were seeded in 96-well plates (10^4^ per well) and incubated with different concentrations of MnCO_3_-ICG for 24 h or 48 h. Cells viability were determined by the MTT assay. The photothermal therapy effect was detected by exposing cells to NIR laser (808 nm, 0.5 W/cm^2^) for 5 min. Cells viability were also determined by the MTT assay.

To visually observe the living and dead cells, 4T1 cells were seeded in six-well plates at a density 10^5^ cells for 24 h. Then, the cells were co-incubated with MnCO_3_-ICG ([Mn]: 5 and 20 μg/mL) for 24 h. The photothermal therapy group were exposed to NIR laser (808 nm, 0.5 W/cm^2^) for 5 min. After that, the cells were continue incubated for another 24 h, and staining with AM/PI dual-staining kit. After washing with PBS for three times, the cells were imaged by an inverted fluorescence microscope.

### The therapy effect of MnCO_3_-ICG under the MCSs

4T1 cells (3000 cells per well) were seeded into 96-wells plate containing 1% agarose to form MCSs. The MnCO_3_-ICG ([Mn]: 20 or 50 μg/mL) were added to the dishes of the MCSs for 24 h. Then, the photothermal therapy group were exposed to NIR laser (808 nm, 0.5 W/cm^2^) for 5 min. The MCSs were continue incubated for another 24 h, and staining with AM/PI dual-staining kit. After washing with PBS for three times, the MCSs were imaged by a laser scanning confocal fluorescence microscope.

### In vivo photothermal and chemodynamic therapy effect

When the tumor reached 60 mm^3^ in average volume, the mice were randomly divide d into 6 groups: (Group 1) PBS (four doses); (Group 2) MnCO_3_-ICG ([Mn]:2 mg/kg, four doses, termed as CDT-1); (Group 3) 2 × MnCO_3_-ICG ([Mn]:4 mg/kg, four doses, termed as CDT-2); (Group 4) 4 × MnCO_3_-ICG ([Mn]:8 mg/kg, four doses, termed as CDT-3); (Group 5) MnCO_3_-ICG ([Mn]:2 mg/kg + L (0.5 W/cm^2^), one dose, termed as PTT); and (Group 6) MnCO_3_-ICG ([Mn]:2 mg/kg + L (0.5 W/cm^2^), three doses, termed as PTT + CDT). The laser (808 nm, 0.5 W/cm^2^, 5 min) was applied to tumor at 4 h post-injection of MnCO_3_-ICG. The body weight and tumor size of the mice were recorded for the next 14 days. The relative tumor volume of each mouse was acquired by dividing by the initial tumor volume Tumor volumes and body weight were also recorded. After 14 days, the mice were euthanized and the collected tumor and major organ were kept for H&E staining.

### Cytokine detection

IL-6 (Elabscience), TNF-α (Elabscience) and IL-1α (Elabscience) in mouse serum samples after different treatment were analyzed with ELISA kits according to the vendors’ protocols.

### Ex vivo analysis of different groups of T cells and dendritic cells (DCs)

To analyze immune cells by flow cytometry, spleens of mice after various treatments were collected and stained according to the manufacturer’s protocols. In brief, to analyze memory T cells, cells from tumors spleens were stained with antibodies against CD8a-APC (BioLegend, catalog no. 100712) and CD3-PE (BioLegend, catalog no. 100206). To analyze the maturation of DCs, cells from tumors spleens were stained with antibodies against CD80-APC (BioLegend, catalog no. 104714), CD86-PE (BioLegend, catalog no. 159204), CD11c-FITC (BioLegend, catalog no. 117306).

### Statistical analysis

The contrast between each group were analyzed by the Student’s t test and the nonparametric test to assess statistical significance (*P < 0.05, **P < 0.01, ***P < 0.001). All experiments were performed at least three times and expressed as means ± SD.

## Results and discussion

### Preparation and characterization of MnCO_3_-ICG complexes

**Fig. 1:**
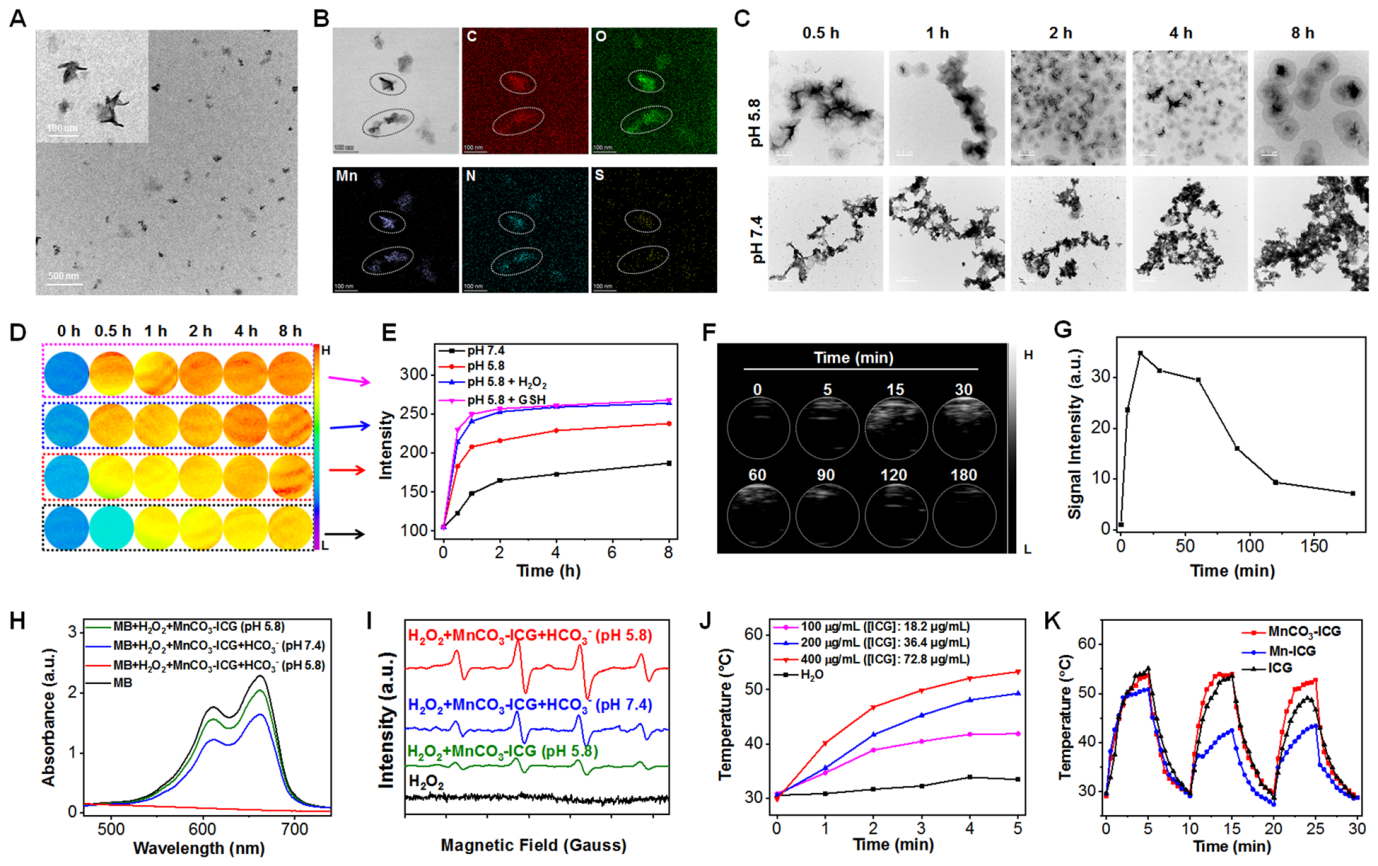
Preparation and characterization of the MnCO_3_-ICG Complexes. **A** TEM imaging of MnCO_3_-ICG complexes. **B** STEM mapping analysis of MnCO_3_-ICG. **C** TEM images of MnCO_3_-ICG after biodegradation in neutral (pH 7.4) and acidic (pH 5.8) PBS for different durations: 0.5, 1, 2, 4 and 8 h. **D** T_1_-weighted images of MnCO_3_-ICG complexes after incubation within different buffer solutions. **E** Time-dependent T_1_ intensity change of MnCO_3_-ICG complexes based on region of interest (ROI) analysis on images from panel **D**. **F** Ultrasound images of the generation of CO_2_ from MnCO_3_-ICG complexes in acidic buffer solution (pH 5.8 + H_2_O_2_) for different time. **G** Ultrasound signal intensity of the generation of CO_2_ based on based on ROI analysis on images from panel **F**. **H** The decline of absorption peak of MB after MnCO_3_-ICG incubated at different buffer solutions. **I** The ESR spectra to detect ·OH produced by MnCO_3_-ICG, using DMPO as the spin trapper. **J** Temperature elevation of H_2_O and different concentrations of MnCO_3_-ICG ([ICG]: 18, 36 and 72 μg/mL) suspensions under continuous irradiation (808 nm, 0.5 W/cm^2^, 5 min). **K** Photothermal heating and natural cooling cycles of ICG, Mn-ICG and MnCO_3_-ICG ([ICG]: 72 μg/mL, 808 nm, 0.5 W/cm^2^)

Non-spherical MnCO_3_-ICG complexes were prepared through a gas diffusion procedure, which are monodisperse with an average diameter of 72 nm (Fig. 1A). The morphology and structure of MnCO_3_-ICG would not change by increasing the feeding ratio of ICG and MnCl_2_·4H_2_O (W/W) (Additional file 1: Fig. S1). Interestingly, spherical Mn-ICG complexes were formed in the absence of the natural decomposition of NH_4_HCO_3_ in an enclosed chamber (Additional file 1: Fig. S1). Elemental mapping indicated that Mn, C, O, N and S were homogeneously distributed in the structure of MnCO_3_-ICG (Fig. 1B). The absorbance spectra of MnCO_3_-ICG showed typical peaks indicating the existence of ICG in the formed MnCO_3_-ICG (Additional file 1: Fig. S2A). With uniform and well-defined structures, the MnCO_3_-ICG prepared at a mass feeding ratio of ICG:MnCl_2_·4H_2_O = 2:50 and reaction time of 2 h in the NH_4_HCO_3_ environment were selected for further experiment (Additional file 1: Fig. S2B). The Mn and ICG contents were determined to be 20.0% and 18.2%, respectively. The stability of complexes played an important role for further bioapplications. Poly(allylamine hydrochloride) (PAH) were modified to increase the stability in physiological environment (Fig. S3A). MnCO_3_-ICG exhibited excellent stability in water, saline solution, fetal bovine serum (FBS), and cell culture medium (DMEM) (Additional file 1: Fig. S3B) and there were no size changes after incubation for 24 h (Additional file 1: Fig. S3C). According to the dispersion and stability of the material, we conduct follow-up evaluation at rate weight of 1:5

### The stimulated response of MnCO_3_-ICG

The realization of a Mn-mediated Fenton-like catalyzed reaction of H_2_O_2_ essentially relies on pH conditions, i.e., the concentration of ${\text{HCO}}_{3}^{ - }$. Therefore, we first studied the release kinetics of MnCO_3_-ICG in biomimetic environment by incubating the material in various pH buffer solutions. TEM data showed that the structures of MnCO_3_-ICG were stable at pH 7.4, but dissociated and re-assembled under mildly acidic condition (Fig. 1C). In the X-ray diffraction (XRD) (Additional file 1: Fig. S4A) and X-ray photoelectron spectroscopy (XPS) (Additional file 1: Fig. S4B–D) analysis of MnCO_3_-ICG and the re-assembled nanoformulation, the structure turned from MnCO_3_ to Mn_3_O_4_, and the valence states of Mn turned from Mn(II) to Mn(IV), after being dissociated and re-assembled. The decomposition profiles of MnCO_3_-ICG were measured using MR and US phantom investigations. Under mildly acidic condition, the Mn^2+^ ion release increased gradually and then increased sharply with existing H_2_O_2_ and GSH, indicating the time-dependent T_1_ intensity change (Fig. 1D, E). Notably, GSH would accelerate the decomposition of MnCO_3_-ICG because GSH can act as a reducing agent and an antioxidant [20, 60]. The US imaging also revealed that the US signal increased sharply after incubation at pH 5.8 and H_2_O_2_, indicating the generation of gas bubbles (Fig. 1F, G). Previous studies revealed that MnO_x_ reacted with H_2_O_2_ under acidic conditions to generate O_2_.[61] To confirm CO_2_ generation, we prepared CaCO_3_ nanoparticles and incubated them under the same conditions [62]. The experimental results of US imaging showed clear bubbles, confirming the generation of CO_2_ (Additional file 1: Fig. S5). Gas chromatogram method was also adopted to study the generation of CO_2_.

### In vitro chemodynamic and photothermal therapy performance

After confirming the generation of Mn^2+^ and ${\text{HCO}}_{3}^{ - }$ under mildly acidic condition, we next evaluated the activity of the Mn-mediated Fenton-like reaction. Several recent discoveries have showed that ${\text{HCO}}_{3}^{ - }$-dependent peroxidation and H_2_O_2_ decomposition catalyzed by Mn^2+^ efficiently amplify intracellular oxidative stress via increasing •OH generation and reducing GSH biosynthesis [32, 63]. Methylene blue (MB) was chosen here as the probe because it can be degraded by •OH. Figure 1H showed a drop in MB absorbance when MB was incubated with MnCO_3_-ICG at pH 5.8 (reduced by 10.6%, green curve); a rapid degradation of MB was found by H_2_O_2_ plus ${\text{HCO}}_{3}^{ - }$ at pH 7.4 (reduced by 28.3%, blue curve) and pH 5.8 (reduced by 99.9%, red curve). We also examined the •OH production capacity of MnCO_3_-ICG by electron paramagnetic resonance (EPR) spectroscopy. In comparison with H_2_O_2_ alone, obvious characteristic peaks (1:2:2:1) were recognized in Mn^2+^/${\text{HCO}}_{3}^{ - }$-mediated Fenton-like reaction group (Fig. 1I). Thus, these results suggested that ${\text{HCO}}_{3}^{ - }$ released from degraded MnCO_3_-ICG played a beneficial role. Furthermore, the absorbance of MB dropped sharply by increasing [${\text{HCO}}_{3}^{ - }$] (Additional file 1: Fig. S6A). Notably, the absorbance of MB completely disappeared after incubation at pH 5.8 in PBS with the same concentration of ${\text{HCO}}_{3}^{ - }$ (red curve in Fig. 1H).

To further confirm that the in situ self-supplying ${\text{HCO}}_{3}^{ - }$-pool accelerates the Mn-mediated Fenton-like reaction, Mn-ICG complexes were also incubated with MB plus H_2_O_2_ at pH 7.4 and 5.8. A significant decrease in MB absorbance was observed in MnCO_3_-ICG (Additional file 1: Fig. S6B). Remarkably, the absorbance of MB degraded by 43.1% after MnCO_3_-ICG incubated for 8 h at pH 5.8 without extra ${\text{HCO}}_{3}^{ - }$ (Additional file 1: Fig. S6C). To further test the importance of the generation of ${\text{HCO}}_{3}^{ - }$, we also removed the ${\text{HCO}}_{3}^{ - }$ (CO_2_) by vacuuming the reactor for 10 min. It was clear that the degradation of MB would decline (Additional file 1: Fig. S7A, B). These results demonstrate that the Mn^2+^ and ${\text{HCO}}_{3}^{ - }$ were generated from MnCO_3_-ICG, and the in situ self-supplying ${\text{HCO}}_{3}^{ - }$-pool accelerated Mn-mediated Fenton-like reaction.

Small organic molecules, including drugs (DOX, EGCG) and photosensitizers (TCPP, ICG) have been described to be able to coordinate with metal ions in recent discoveries [59, 62, 64–67]. Our MnCO_3_-based nanocomplexes use the clinically approved fluorescent dye ICG; it has value in both photodynamic therapy and photothermal therapy [68, 69]. The MnCO_3_-ICG showed strong absorption at 808 nm indicating that ICG was successfully incorporated into the ICG structure (Additional file 1: Fig. S8). MnCO_3_-ICG solutions ([ICG]: 18.2, 36.4 and 72.8 μg/mL) were exposed to an 808 nm laser (0.5 W/cm^2^), and the maximum temperature can reach up to 53.3 °C within 5 min (Fig. 1J). No obvious photobleaching of MnCO_3_-ICG was observed after exposure to 808 nm laser (0.5 W/cm^2^) for three laser on/off cycles compared with Mn-ICG and free ICG (red curve in Fig. 1K). Interestingly, Mn-ICG showed obvious photobleaching than that of free ICG (blue curve in Fig. 1K).

### In vitro evaluation of the therapeutic effect of MnCO_3_-ICG

**Fig. 2:**
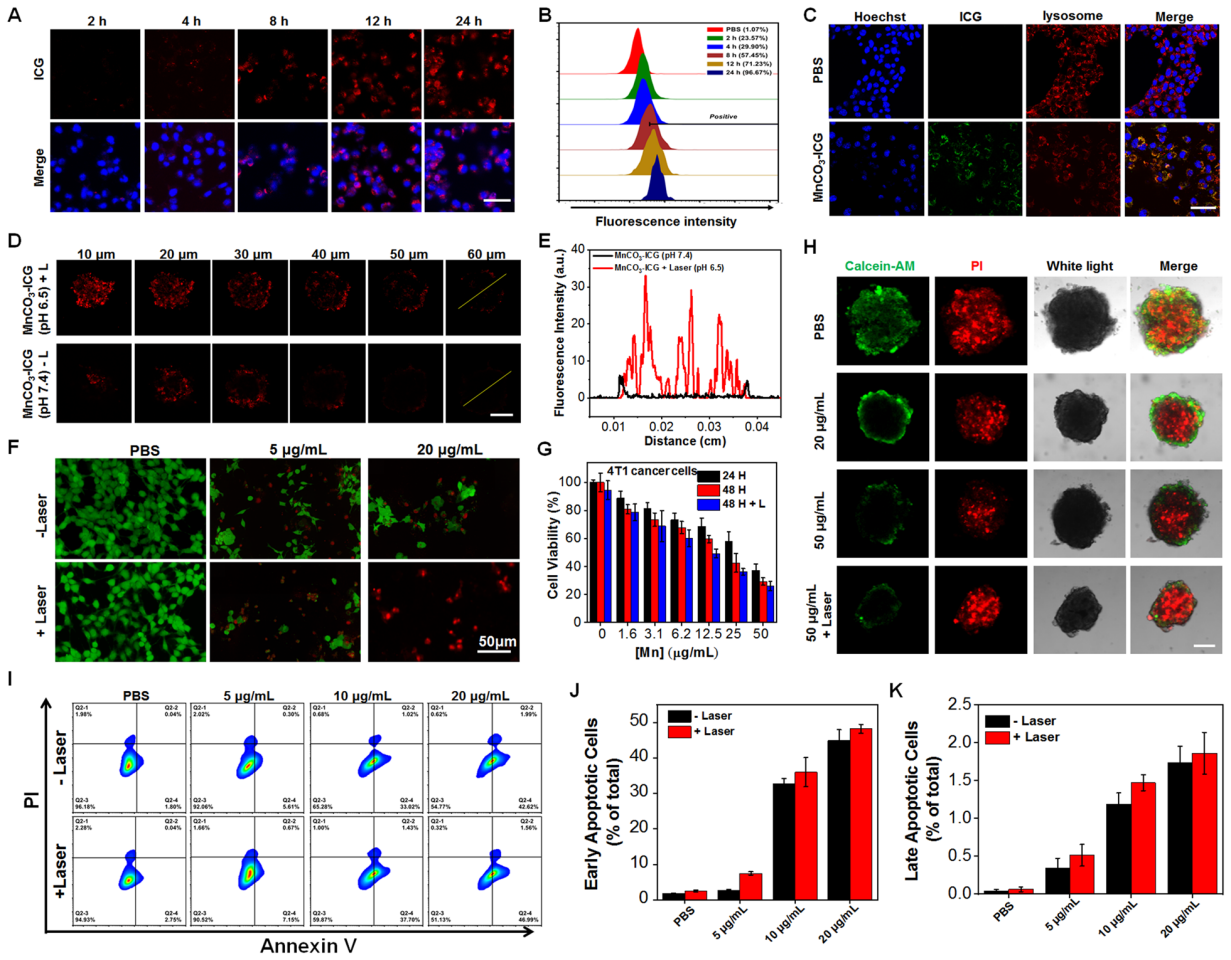
The Efficiently Induce Tumor Cell Death by MnCO_3_-ICG. **A** Cell uptake of 4T1 cells incubated with MnCO_3_-ICG at different time (scale bar, 50 μm). **B** Flow cytometric and quantitative analyses of internalization of MnCO_3_-ICG at different time. **C** CLSM evaluation on the lysosomal escape of MnCO_3_-ICG. The blue, green, and red colors indicate cell nucleus, ICG, and lysosome, respectively (scale bar, 50 μm). **D** CLSM imaging of the MnCO_3_-ICG penetration after different treatment (pH 6.5 with laser or pH 7.4 without laser) in MCSs (scale bar, 200 μm). **E** Corresponding fluorescence profiles in **D**. **F** Calcein-AM and PI co-stained 4T1 cells with different concentration of MnCO_3_-ICG with or without 808 nm laser (0.5 W/cm^2^, 5 min) irradiation, the green and red fluorescence indicate live cells and dead cells. **G** Cell viabilities of 4T1 cells measured by MTT assays, after incubating with different concentration of MnCO_3_-ICG with or without 808 nm laser (0.5 W/cm^2^, 5 min) irradiation. **H** Calcein-AM and PI co-stained 4T1 MCSs after different treatment for three days (PBS, MnCO_3_-ICG with [Mn]: 20 μg/mL, 50 μg/mL without and with laser irradiation (808 nm, 0.5 W/cm^2^, 5 min)). **I** Flow cytogram representing apoptosis assay based on Annexin V-FITC and propidium iodide staining of 4T1 cells after treatment with different therapeutic groups. **J** Early apoptosis (V + /PI −) and (**K**) late apoptosis (V + /PI +) of the 4T1 cells after treatment with different therapeutic groups

We next studied the cellular internalization profile of MnCO_3_-ICG in murine 4T1 breast cancer cells by using confocal microscopy. By recording the fluorescence of ICG, clear red fluorescence signals were observed in the cytoplasm by prolonging the incubation time (Fig. 2A). The uptake profile was also verified by flow cytometry (Fig. 2B). Notably, such MnCO_3_-ICG exhibited time-dependent cell internalization profiles via endocytosis pathways as evidenced by the colocalization of the fluorescence of ICG fluorescence with that of LysoTracker (Fig. 2C). The colocalization of ICG and lysosome at 24 h post-incubation was 0.46 ± 0.050 by Pearson’s correlation via ImageJ analysis. To find out whether the MnCO_3_-ICG might result in enhanced penetration, we used the multicellular spheroids (MCSs) derived from 4T1 cells as an in vitro model. The MCSs were treated with MnCO_3_-ICG under different incubation environment (pH 6.5 with laser or pH 7.4 without laser) for 24 h, and the penetration process was measured by using confocal microscopy. MnCO_3_-ICG irradiated by laser under slightly acidic environment leaded to better cellular uptake, when compared to control group (Fig. 2D, E).

The in vitro anti-tumor efficiency of MnCO_3_-ICG was evaluated in multiple ways, including calcein-AM and propidium iodide (PI) staining, 3-(4,5-dimethylthi-azol-2-yl)-2,5-diphenyl-2*H*-tetrazolium bromide (MTT) assay, flow cytometry and MCSs. As a result, we found that treatment of only MnCO_3_-ICG results in significant concentration-dependent cell death as indicated by the live-dead dual staining using calcein-AM and PI; the most effective cell death was induced by the treatment of MnCO_3_-ICG plus laser exposure (Fig. 2F), which was confirmed by the existence of only red fluorescence in the cells. As expected, MnCO_3_-ICG displayed a dose- and time-dependent toxicity to 4T1 cells. Lower concentrations of MnCO_3_-ICG had only minor harm to 4T1 cells, and higher concentrations of MnCO_3_-ICG demonstrated a rapid increase in cytotoxicity; laser exposure could further enhance the cytotoxicity (Fig. 2G). Flow cytometry apoptosis technique was also applied to precisely analyze cell apoptosis rate (Fig. 2I–K). Furthermore, we evaluated anticancer efficacy against 4T1 3D MCSs by AM-PI staining in vitro (Fig. 2H). A decreasing number of live cells (represented by green color) and a reduced size of 3D MCSs was observed in MnCO_3_-ICG and MnCO_3_-ICG with laser group, which indicated the excellent anti-cancer efficacy. These results could be explained by excessive ROS-induced oxidative stress. Note that the remarkable killing effect was not limited by tumor types because the MnCO_3_-ICG could efficiently destroy multiple types of human tumor cells, including liver cancer and glioma (Additional file 1: Fig. S9A, B); normal human cells managed to tolerate the adverse influence of MnCO_3_-ICG with much higher cell viabilities than tumor cells under the same treatment (Additional file 1: Fig. S10). This higher tolerance was attributed to the presence of a sufficient amount of catalase to prevent the normal cells from entering an oxidative stress state caused by excessive ROS [70, 71].

To compare the treatment efficacy, we next calculated the half maximal inhibitory concentration (IC_50_) of different treatments and tumor cellular lines. Using 4T1 as an example, we found the IC_50_ of MnCO_3_-ICG (11.2 ± 1.0 μg/mL) to be 5.8-fold lower than that of Mn-ICG (64.5 ± 1.3 μg/mL). The results confirmed that in situ self-generation of ${\text{HCO}}_{3}^{ - }$, The decomposition of MnCO_3_-ICG, is an important and indispensable condition for Mn-mediated Fenton-like reactions. This in turn induces enhanced chemodynamic therapy.

### In vitro oxidative stress generation of MnCO_3_-ICG

**Fig. 3:**
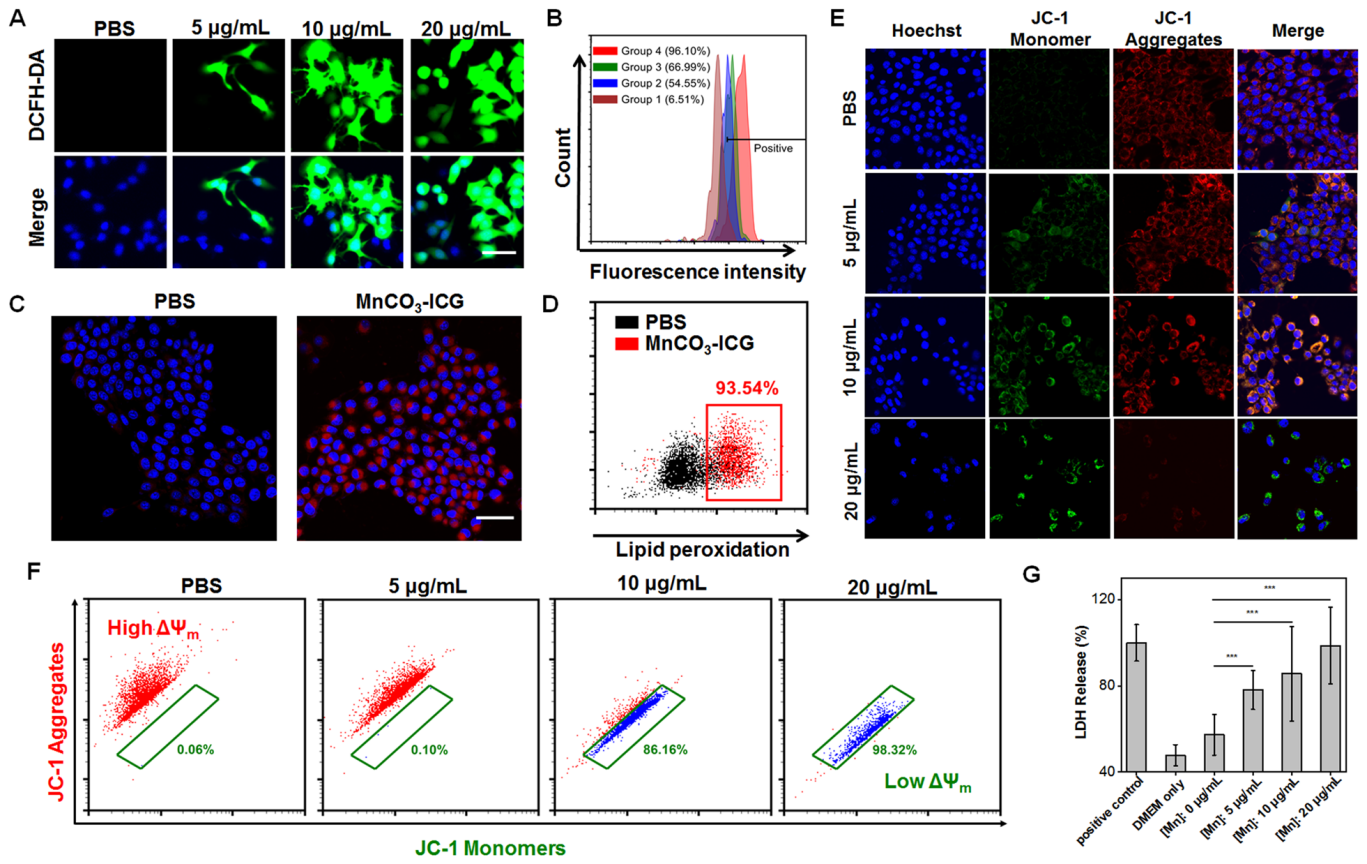
In vitro Oxidative Stress Generation of MnCO_3_-ICG. **A** Intracellular ·OH generation detected by DCFH-DA probe (scale bar, 30 μm). **B** Flow cytometry data for DCFH-DA probe treated with Group 1: PBS, Group 2: MnCO_3_-ICG ([Mn]: 5 μg/mL), Group 3: MnCO_3_-ICG ([Mn]: 10 μg/mL), Group 4: MnCO_3_-ICG ([Mn]: 20 μg/mL). **C** CLSM observation on the intracellular distribution of lipoperoxides in 4T1 cells after incubation with PBS and MnCO_3_-ICG for 24 h. The red fluorescence is the lipid ROS in cells and membranes after the staining with BODIPY-C11 (scale bar, 50 μm). **D** Flow cytometric and quantitative analyses of lipid peroxidation. **E** CLSM observation on the changes in the mitochondrial membrane potential of 4T1 cells after incubation with different concentration of MnCO_3_-ICG. The blue, red, and green colors indicate cell nucleus, and JC-1J-aggregates and monomer, respectively (scale bar, 50 μm). **F** ΔΨ_m_ was assessed by detecting the red fluorescence and green fluorescence via flow cytometer. **G** LDH release assay after incubation with different concentration of MnCO_3_-ICG (***p < 0.001)

Because of the higher efficacy of anti-tumor efficiency of MnCO_3_-ICG, we next investigated the cellular ROS production and anticancer effect in vitro. 4T1 cancer cells were incubated with various concentrations of MnCO_3_-ICG, and then stained with DCFH-DA (ROS indicator). The confocal images of 4T1 cells showed that those cells treated with a high concentration of MnCO_3_-ICG showed much stronger green fluorescence of DCFH-DA, suggesting a concentration-dependent ROS generation induced by MnCO_3_-ICG (Fig. 3A). Interestingly, with the same incubation time and concertation of Mn in Mn-ICG, we observed obviously weak green fluorescence of DCFH-DA (Additional file 1: Fig. S11). These further confirmed that the in situ self-generated ${\text{HCO}}_{3}^{ - }$ ions from the decomposition of MnCO_3_-ICG accelerated the Mn-catalyzed decomposition of H_2_O_2_ and peroxidation reactions [5, 27]. For instance, at a concentration of 20 μg/mL of Mn^2+^, quantitative flow cytometry showed that the fluorescence of DCFH-DA in MnCO_3_-ICG was 14.7-fold higher than that of PBS group (Fig. 3B).

The intracellular lipid peroxidation levels were evaluated using BODIPY™ 581/591 C11 (lipid peroxidation sensor) and malondialdehyde (MDA) assay kits. After treatments, the levels of lipid peroxidation increased to 145.0 ± 5.1%, and 101.8 ± 16.7% (Additional file 1: Fig. S12) for MnCO_3_-ICG ([Mn]: 20 μg/mL) and PBS, respectively. The increase of fluorescence in the plasma membranes revealed that incubation of 4T1 cells with MnCO_3_-ICG successfully led to lipid peroxidation (Fig. 3C). Quantitative analysis showed that the fluorescence of BODIPY-C11 in the 4T1 cells after incubation with MnCO_3_-ICG was 1.5- and 3.0-fold higher than that of Mn-ICG and cells only (Additional file 1: Fig. S13), respectively. Actually, 93.5% lipid peroxide efficiency was confirmed by flow cytometry (Fig. 3D). These observations were immediate evidence that the in situ self-supplying bicarbonates efficiently amplified intracellular oxidative stresses in a Mn-mediated Fenton-like reaction using MnCO_3_-ICG.

The mitochondria are a primary site of energy production and the site of apoptosis. Mitochondrial dysfunction is a distinctive feature of apoptosis, including loss of mitochondrial membrane potential [72]. Consequently, we measured the changes in the mitochondrial membrane potential of tumor cells using a JC-1 assay kit, in which red fluorescence indicates healthy mitochondria and green fluorescence indicates mitochondria in poor health [73]. According to the CLSM images (Fig. 3E), the red fluorescence was sharply switched to green when increasing the concentration of MnCO_3_-ICG, and completely switched to green at the highest concentration of Mn (*i.e.* 20 μg/mL). Flow cytometric analyses also revealed rapid decrease of mitochondrial membrane potential by MnCO_3_-ICG (Fig. 3F). In comparison, there was little red fluorescence that switched to green in Mn-ICG (Additional file 1: Fig. S14). Lactate dehydrogenase (LDH) is an intracellular active enzyme and was released into the cell culture medium [74, 75], indicating that the cell walls were breached (Fig. 3G; Additional file 1: Fig. S15) [76]. These data suggest a highly negative mitochondrial membrane potential due to the introduction of bicarbonates that induce amplified intracellular oxidative stresses of Mn species [77].

### In vivo biodistribution and MR/FL-dual mode imaging

**Fig. 4:**
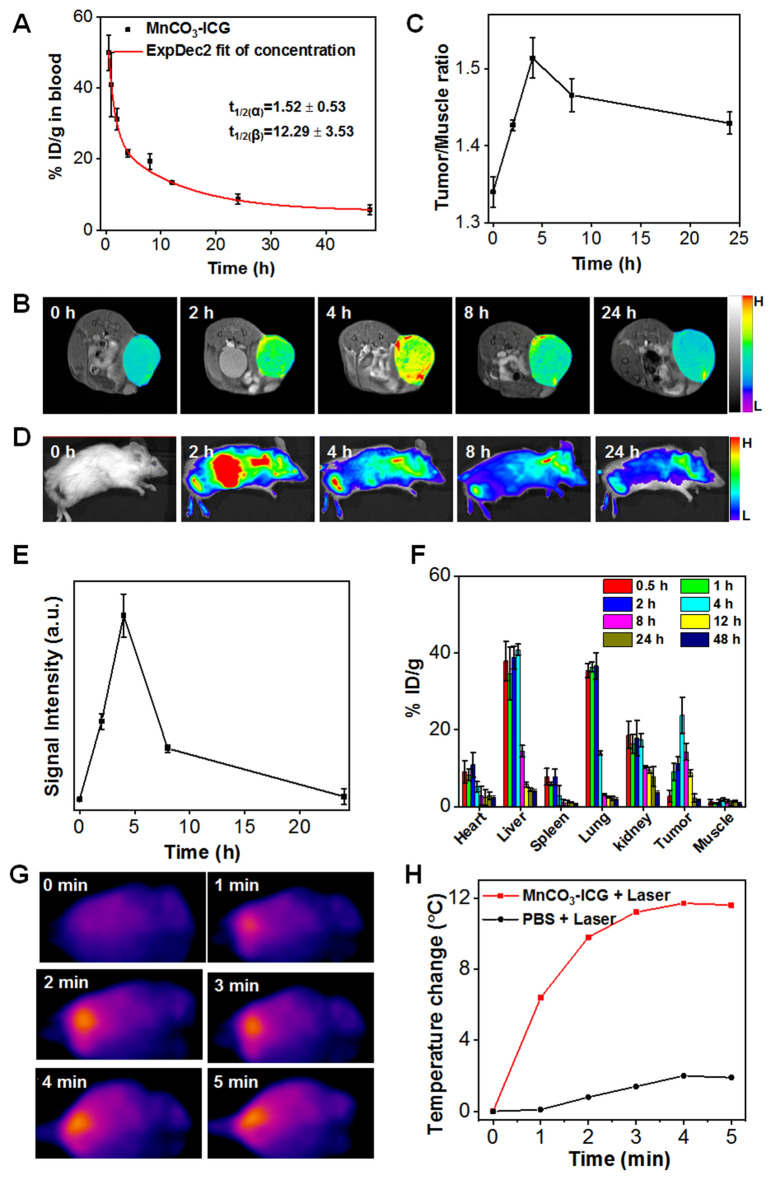
The Distribution of the MnCO_3_-ICG Complexes on 4T1 Tumor-bearing Mice. **A** Time course of blood levels of MnCO_3_-ICG levels following intravenous injection. **B** In vivo MRI images and (**D**) fluorescence images of BALB/C tumor-bearing mice taken at different time points after injection of MnCO_3_-ICG. **C** Quantification analysis of MRI signal change in tumor/muscle based on region of interest (ROI) analysis on images from panel **B**. **E** Quantification analysis of the tumor ratio of fluorescence signal change in tumors based on ROI from panel **D**. **F** Biodistribution of Mn (% injected dose (ID) Mn per gram tissue) in main tissues and tumors after intravenous administration of MnCO_3_-ICG. **G** Thermal images and (**H**) real-time temperature curve of BALB/C tumor-bearing mice treated with MnCO_3_-ICG and 808 nm laser (0.5 W/cm^2^) irradiation

The in vivo pharmacokinetic profiles of MnCO_3_-ICG ([Mn]: 2 mg/kg) were carefully assessed by detecting the distribution of Mn in 4T1 tumor-bearing mice. Blood circulation profiles followed a classical two-compartment model with the first half-time and second half-time determined to be 1.5 ± 0.5 h and 12.3 ± 3.5 h, respectively (Fig. 4A). We found that MnCO_3_-ICG passively accumulated into the tumor area and reached the highest level at 4 h post-injection under a 9.4 T in vivo MRI scanner system (Fig. 4B, C). The results showed a relatively high longitudinal relaxivity (*r*_1_ = 4.3 mM^−1^ s^−1^) (Additional file 1: Fig. S16). In addition, fluorescence imaging was also conducted on 4T1 tumor mice, and showed similar results (Fig. 4D, E). By detecting the Mn in organs using ICP-MS, we found the maximum tumor accumulation of MnCO_3_-ICG to be 23.7 ± 4.7%ID/g (percent injected dose per gram tissue). This remained as high as 8.8 ± 0.9%ID/g at 12 h post-injection (Fig. 4F). The ex vivo fluorescence imaging (Additional file 1: Fig. S17) at 24 after intravenous injection showed an obvious signal in the tumor. The signal intensity of MR and fluorescence in tumor regions remained strong even at the 24 h post injection, which indicated the accumulation of Mn^2+^ into the tumor site for a long time to offer desirable and long-term CDT efficacy. At 4 h post-injection when the accumulation reached the highest level, we irradiated the tumor area with 808 nm laser (0.5 W/cm^2^). The temperature increased sharply by about 10 °C within 2 min and remained at that temperature for 5 min (Fig. 4G, H).

**Fig. 5:**
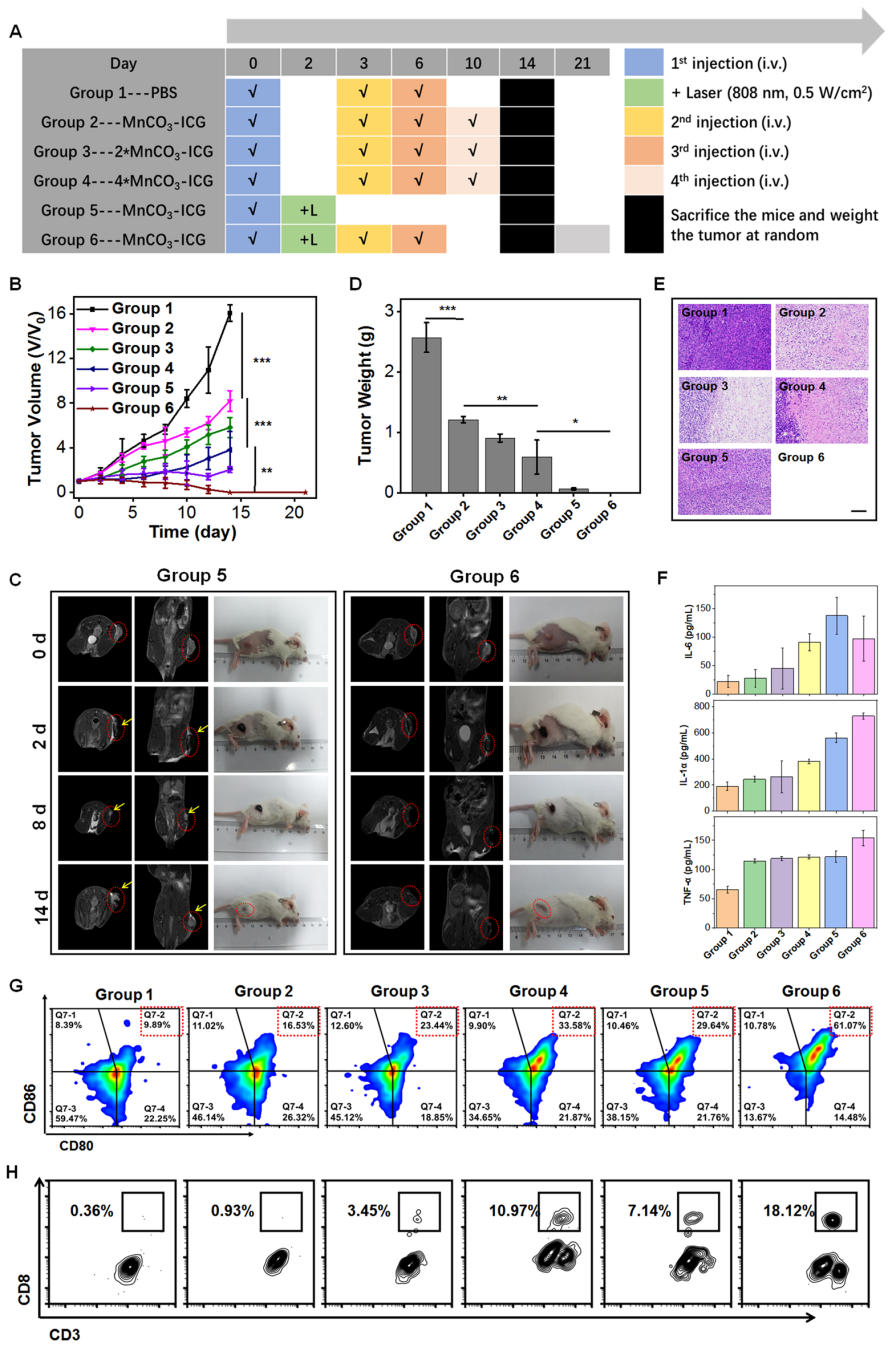
In vivo therapeutic efficacy and immunity response of the MnCO_3_-ICG complexes on 4T1 Tumor-bearing Mice. **A** Therapy approach for tumor-bearing mice. **B** Tumor growth curves of BALB/C tumor-bearing mice after various treatments (n = 5) (**p < 0.01; ***p < 0.001). **C** Final tumor weights of BALB/C tumor-bearing mice exposed to different formulations after the different treatment (*p < 0.05; **p < 0.01; ***p < 0.001). **D** Hematoxylin & eosin (H&E)-stained tumor sections from BALB/C tumor-bearing mice after various treatments (scale bar, 100 μm). **E** The T_2_-MR imaging and digital photos of mice through the treatment period (group 5 and group 6). **F** IL-6, IL-1α and TNF-α in serum obtained from immunized mice. Flow cytometric analyses of the populations of (**G**) matured DC cells and (**H**) CD8 + T cells and CD3 + T cells in splenocytes of mice immunized after the different treatment

Next, we carefully evaluated the in vivo therapeutic efficacy of MnCO_3_-ICG with or without laser irradiation by using 4T1 tumor mice. When the tumor volume reached about 60 mm^3^, the mice were divided into six groups (n = 5/group) (Fig. 5A): (Group 1) PBS (four doses); (Group 2) MnCO_3_-ICG ([Mn]:2 mg/kg, four doses, termed as CDT-1); (Group 3) 2 × MnCO_3_-ICG ([Mn]:4 mg/kg, four doses, termed as CDT-2); (Group 4) 4 × MnCO_3_-ICG ([Mn]:8 mg/kg, four doses, termed as CDT-3); (Group 5) MnCO_3_-ICG ([Mn]:2 mg/kg + L (0.5 W/cm^2^), one dose, termed as PTT); and (Group 6) MnCO_3_-ICG ([Mn]:2 mg/kg + L (0.5 W/cm^2^), three doses, termed as PTT + CDT). The laser (808 nm, 0.5 W/cm^2^, 5 min) was applied to the tumors at 4 h post-injection of MnCO_3_-ICG. We found that the tumors on the mice of Groups 4, 5 and 6 were more effectively suppressed over those in the other Groups (Fig. 5B; Additional file 1: S18). The tumors in Group 5 recurred towards the ending point, however, there was no recurrence even after 21 days post-treatment in Group 6 (Fig. 5B, C).

### In vivo therapeutic efficacy of MnCO_3_-ICG for subcutaneous tumor

To evaluate the therapy efficacy, we sacrificed all mice in Groups 1–5 and randomly selected two mice in Group 6 to measure the tumor weight. The weights and photographs were consistent with the statistical analysis of tumor size (Fig. 5C; Additional file 1: Fig. S19). The statistical analysis showed that the tumor growth inhibition (TGI) rates were 49.0% (Group 2), 63.8% (Group 3), and 76.3% (Group 4) (Fig. S20). Remarkably, Group 6 resulted in a complete tumor inhibition (Fig. S20 and S21). The hematoxylin and eosin (H&E) images (Fig. 5D) and immunofluorescence staining of vessel (Additional file 1: Fig. S22) proved that the tumor in the PTT group had a complete tumor tissue and vessel structure, which was prerequisite and foundation of MnCO_3_-ICG accumulation to the tumor after irradiation. Furthermore, the H&E staining of tumor slices collected from the different treatment mice exhibited the most severe histological damages (i.e. no tumor modules were found) versus those with no treatment (Fig. 5D). Hard scabs were formed after thermal therapy, and the tumor sizes were hard to measure by a caliper; thus, MRI was employed to monitor the tumor development after the laser irradiation. The MRI of Groups 5 and 6 indicated rapidly shrunk tumor volumes during 2–12 days (Fig. 5E); Group 5 had an obvious recurrence, which is one of the most common issues in photothermal therapy (Fig. 5E). Notably, there was no tumor modules to be found in Group 6 up to 21 days post-treatment (Additional file 1: Fig. S21), which is consistent with the H&E staining (Fig. 5D). Furthermore, ROS staining and TUNEL staining (Additional file 1: Fig. S23) of tumor slices further showed that tumor cells were serious damaged by MnCO_3_.

### In vivo immunogenic effect evaluation of combination therapy strategy

We speculated that the 100% tumor growth inhibition was a result of CDT&PTT induced immunogenic cell death combined with novel adjuvants (Mn^2+^) activating innate immunity [17, 54, 55]. Herein, to further elucidate the enhanced anti-tumor immunity afforded by Mn^2+^ adjuvant strategy, the in vivo immunogenic effect was evaluated by testing the dendritic cells (DCs) maturation and T cells activation of mice spleen after treatment, as well as the major cell cytokines in serum from a portion of the tested mice. In addition, the dramatic changes of IL-6, IL-1α and TNF-α in serum were observed after different treatments (Fig. 5F), which indicated MnCO_3_-ICG-mediated acute inflammatory response. As shown in Fig. 5G, the percentage of mature DCs in group 6 (PTT & CDT) was much higher than that in the control. Compared with mice treated with CDT alone or PTT alone, combination therapy strategy showed lots of CD8^+^ cytotoxic T lymphocytes (CTLs) recruitment, indicating the effective activation of innate immune response (Fig. 5H; Additional file 1: Fig. S24). These results indicated that MnCO_3_-ICG had a potential to activate immunotherapeutic efficacy, due to the use of immune adjuvants.

### In vivo safety evaluation of MnCO_3_-ICG complexes

The biocompatibility of MnCO_3_-ICG was evaluated by recording the body weight of treated mice throughout the entire treatment process and the histological examinations of main organs at day 14 using H&E staining. We found that the body weight of these mice showed negligible fluctuations (Additional file 1: Fig. S25). Moreover, no obvious disturbance was found in the main organs including the liver, spleen, kidneys, heart, and lung (Additional file 1: Fig. S26). Collectively, these therapeutic studies in vivo demonstrate that MnCO_3_-mediated amplification of intracellular oxidative stress is an effective anticancer strategy with few biological side effects.

### In vivo therapeutic efficacy of MnCO_3_-ICG for orthotopic hepatocellular carcinoma

**Fig. 6:**
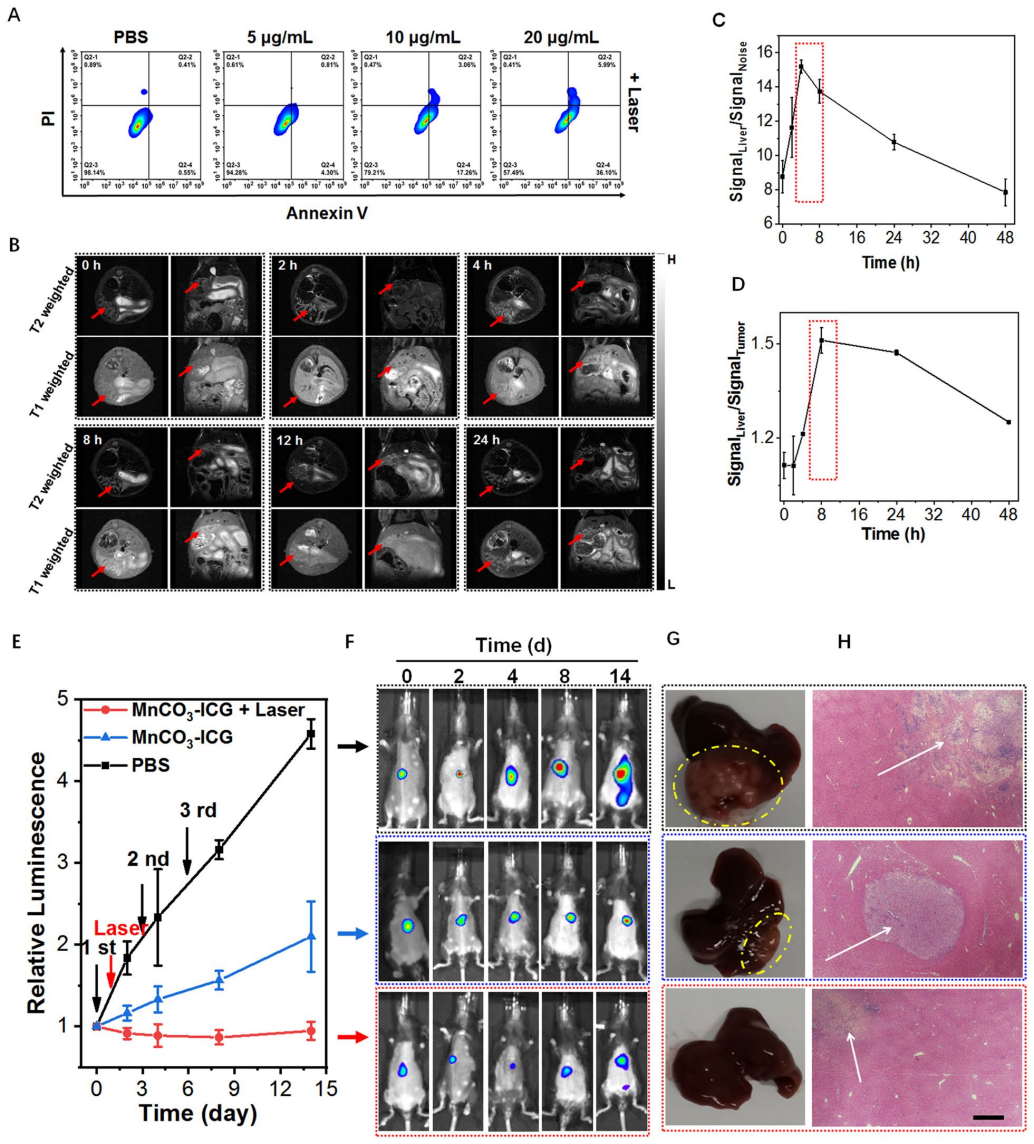
The Antitumor Efficiency for Hepatocellular Carcinoma (Hep 1–6) Cells and Orthotopic Hepatic Tumors. **A** Flow cytogram representing apoptosis assay based on Annexin V-FITC and propidium iodide staining of 4T1 cells after treatment with different therapeutic groups. **B** In vivo T_1_/T_2_ MRI images for orthotopic hepatic tumor-bearing mice taken at different time points after injection of MnCO_3_-ICG. Quantification analysis of MRI signal change in **C** tumor/noise and (**D**) tumor/liver based on region of interest (ROI) analysis on images from panel **B**. **E** Relative luminescence intensity changes based on the bioluminescence images (BLI). **F** BLI changes after different treatments duration of therapy. **G** Photographs of representative tumors and (**H**) H&E-stained tumor sections taken from tumor-bearing mice after various treatments (scale bar: 200 μm)

Due to the excellent therapeutic effect and immune response, we further evaluated the anticancer activity of MnCO_3_-ICG in an orthotopic hepatocellular carcinoma (Hep 1–6) model. Flow cytometry assay revealed a significant dose-dependent increase of the Hep 1–6 cell apoptosis upon treatment (Fig. 6A). We also evaluated the oxidative stress response of Hep 1–6 tumor cells through the generation of ROS (Additional file 1: Fig. S27) and the level of lipid peroxidation (Additional file 1: Fig. S28). The obvious increase of fluorescence signal indicating MnCO_3_-ICG can induce sharp cellular oxidative stress. Mitochondrial and cell membrane damage induced by oxidative stress was confirmed by JC-1 assay (Additional file 1: Fig. S29) and LDH assay kit (Additional file 1: Fig. S30).

Therefore, orthotopic hepatocellular carcinoma therapy of MnCO_3_-ICG was conducted on Hep 1–6-tumor-bearing mice. To minimize photothermal-mediated normal liver tissue damage, distributing pattern of MnCO_3_-ICG between normal liver and tumor was studied using magnetic resonance imaging. The MR signal intensity in liver and tumor gradually increased after MnCO_3_-ICG injection (Fig. 6B). There were significant different signal changes between normal liver and tumor after treatment for 8 h, although the hepatic uptake was not the highest at this time (Fig. 6C, D). The therapeutic efficacy of MnCO_3_-ICG was evaluated after confirming the establishment of tumors by bioluminescence imaging (BLI). The tumor-bearing mice were divided randomly into 3 groups (n = 5/group): (1) PBS; (2) MnCO_3_-ICG (2 mg/kg, three doses, termed as CDT); (3) MnCO_3_-ICG ([Mn]:2 mg/kg + L (0.5 W/cm^2^), three doses, termed as PTT + CDT). Three injections were administered intravenously on days 0, 3 and 6, and the laser (808 nm, 0.5 W/cm^2^, 5 min) was applied to the tumors at 8 h post-1st-injection of MnCO_3_-ICG. During the treatment, the orthotopic hepatic tumor growth was monitored by BLI. The fluorescence intensity of group 3 remained unchanged or even decreased slightly, which was a sharp contrast to the increase of fluorescence intensity in the control group (Fig. 6E). Combination immunotherapy strategy significantly inhibited tumor growth (TGIs on day 12 were 54.2% (Group 2) and 79.4% (Group 3)). To further study the therapeutic efficacy, livers on mice after various treatments were harvested, photographed, and analyzed by H&E staining (Fig. 6F–H). Significant neoplasms were observed in the liver tissue of the control group. Severely damaged structural disruptions and pathological changes were observed in tiny nodules of tumor after treatment. H&E staining of livers indicating the safety of MnCO_3_-ICG, even with the laser for hepatocellular carcinoma in situ. In brief, these results demonstrated that MnCO_3_-ICG-mediated PTT&CDT combined immunotherapy strategy could serve as a potential therapy for the elimination of tumor in situ.

## Conclusion

In summary, we proposed a self-supplying bicarbonate- and Mn-pool Fenton-like MnCO_3_-ICG catalyst with amplification of tumor oxidative stress and regulation of intracellular pH. ICG molecules were employed as coordination sites to regulate the decomposition level in the acidic environment. MnCO_3_-ICG degraded and released ${\text{HCO}}_{3}^{ - }$ and Mn^2+^ in situ, and the increased ${\text{HCO}}_{3}^{ - }$ inside the cells could escalate Mn-mediated Fenton-like reaction to accelerate the generation of •OH for oxidative stress damage of tumors cells. The evaluation of cellular internalization in vitro showed that MnCO_3_-ICG could be internalized by cells and released from lysosome to cytosols, in which Mn^2+^ had more opportunities to generate cellular oxidative stress by reacting with H_2_O_2_ in the presence of ${\text{HCO}}_{3}^{ - }$. The in vivo results confirmed that the combination of CDT, PTT combined with nanoadjuvant (Mn^2+^)-enhanced immunotherapy effectively suppressed the tumor growth, and realized complete tumor elimination. The combination therapy strategy promotes DC maturation, enhances the CTL-mediated cytotoxic effect, and generates multiple pro-inflammatory factors (IL-6, IL-1α and TNF-α). This study provides a potential synergistical strategy to improve the therapeutic effect for orthotopic tumors by releasing “ion drug” in an on-demand manner.

## Supplementary Information

**Additional file 1: Figure S1.** Typical TEM images of MnCO_3_-ICG complexes prepared at different feeding ratios of ICG and MnCl_2_·4H_2_O. **Figure S2.** (a) the absorption spectra of MnCO_3_-ICG complexes at different time. (b) Typical TEM images of MnCO_3_-ICG complexes prepared at different time. **Figure S3.** (a) TEM imaging of MnCO_3_-ICG-PAH complexes prepared with different feeding ratios of MnCO_3_-ICG and PAH. (b) Photographs of MnCO_3_-ICG-PAH complexes after being incubated in H_2_O, PBS (7.4), cell medium (DMEM), and serum (FBS) for 12 h. (c) The hydrodynamic diameter of MnCO_3_-ICG-PAH complexes with different feeding ratios. **Figure S4.** (a) X-ray powder diffraction pattern of the MnCO_3_-ICG and the re-assembled nanoformulation after incubation in the acidic buffer solutions. (b) XPS spectra for MnCO3-ICG and the re-assembled nanoformulation after incubation in the acidic buffer solutions. XPS spectra of Mn2p for (c) for MnCO_3_-ICG and (d) the re-assembled nanoformulation after incubation in the acidic buffer solutions. **Figure S5.** (a) Ultrasound images of the generation of CO_2_ after CaCO_3_ incubation with different time (pH 5.8 + H_2_O_2_). (b) Ultrasound signal intensity of the generation of CO_2_ after CaCO_3_ incubation with different time (pH 5.8 + H_2_O_2_). (c) The typical chromatograms of CO_2_ generated after MnCO_3_-ICG incubation with acidic environment. **Figure S6**. (a) Colorimetric analysis of the Fenton-like reaction for MB decolorization with different concentration of HCO_3_− in acidic environment. (b) Colorimetric analysis of the Fenton-like reaction for MB decolorization with different treatment. (c) Colorimetric analysis of the Fenton-like reaction for MB decolorization after different reaction time with acidic environment (pH 5.8). **Figure S7.** (a) Colorimetric analysis of the Fenton-like reaction for MB decolorization after pulling a vacuum. (b) Bar plot showing the of MB after different treatments. **Figure S8.** The absorption spectra of ICG, Mn-ICG and MnCO_3_-ICG. **Figure S9.** (a) Hep G2 cells and (b) U87MG cells incubated with different concentrations of MnCO_3_-ICG with or without 808 nm laser (0.5 W/cm^2^, 5 min) irradiation. **Figure S10**. Relative cellular viabilities of normal L02 and 3T3 cells incubated with different concentrations of MnCO_3_-ICG. **Figure S11**. Intracellular ·OH generation after incubation with Mn-ICG detected by DCFH-DA probe (scale bar, 50 μm). **Figure S12**. Lipid damage assessment measured by lipid peroxidation assays (*p < 0.05). **Figure S13**. (a) CLSM observation on the intracellular distribution of lipoperoxides in 4T1 cells after incubation with PBS and Mn-ICG for 24 h. The red fluorescence is the lipid ROS ROS in cells and membranes after the staining with BODIPY-C11 (scale bar, 50 μm). (b) lipoperoxides, based on BODIPY staining resuts in panel (a). **Figure S14**. (a) CLSM observation on the changes in the mitochondrial membrane potential of 4T1 cells after incubation with different concentration of Mn-ICG (scale bar, 50 μm). (b) The membrane potential (ΔΨm) changes, assessed by JC-1staining. **Figure S15**. LDH release assay after incubation with different concentration of Mn-ICG. **Figure S16**. T_1_-relaxation rate (r_1_) and T_1_-weighted MR images of MnCO_3_-ICG. **Figure S17.** Ex vivo fluorescence images of the organs harvested in BALB/C tumor-bearing mice at 24 h post-injection. **Figure S18.** Body photos in different formulations after the 14-day treatment period. Groups 1, 2, 3 and 4 were used to represent PBS, MnCO_3_-ICG ([Mn]: 2 mg/kg, four dose), 2 × MnCO_3_-ICG ([Mn]: 4 mg/kg, four dose), 4 × MnCO_3_-ICG ([Mn]: 8 mg/kg, four dose), respectively. **Figure S19.** Images of representative tumors taken from mice in different formulations after the 14-day treatment period. Groups 1, 2, 3, 4, 5 and 6 were used to represent PBS, MnCO_3_-ICG ([Mn]: 2 mg/kg, four dose), 2 × MnCO_3_-ICG ([Mn]: 4 mg/kg, four dose), 4 × MnCO_3_-ICG ([Mn]: 8 mg/kg, four dose), MnCO_3_-ICG ([Mn]: 2 mg/kg + L (0.5 W/cm^2^), one dose) and MnCO_3_-ICG ([Mn]: 2 mg/kg + L (0.5 W/cm^2^), three dose), respective. **Figure S20**. The tumor growth inhibition curves of BALB/C tumor-bearing mice exposed to different formulations after the treatment period. Groups 1, 2, 3, 4, 5 and 6 were used to represent PBS, MnCO_3_-ICG ([Mn]: 2 mg/kg, four dose), 2 × MnCO_3_-ICG ([Mn]: 4 mg/kg, four dose), 4 × MnCO_3_-ICG ([Mn]: 8 mg/kg, four dose), MnCO_3_-ICG ([Mn]: 2 mg/kg + L (0.5 W/cm^2^), one dose) and MnCO_3_-ICG ([Mn]: 2 mg/kg + L (0.5 W/cm^2^), three dose), respective. **Figure S21**. The T2-MR imaging and digital photos of mice BALB/C tumor-bearing after the 21-day treatment period (group 6). Groups 6 was used to represent MnCO_3_-ICG([Mn]: 2 mg/kg + L (0.5 W/cm^2^), three dose), respectively. **Figure S22.** Representative immunofluorescence staining of nucleus (blue) and vessel (green) on the tumor slices collected 14-day after laser irradiation (scale bar, 50 μm). **Figure S23.** Representative immunofluorescence staining of TUNEL (nucleus (blue) and apoptotic cells (red)) and ROS (nucleus (blue) and ROS (green)) on the tumor slices. (scale bar, 200 μm). Figure S24. The flow cytometric histograms showing the intratumor infiltration of the effector T cells (CD8 + T cells). Groups 1, 2, 3, 4, 5 and 6 were used to represent PBS, MnCO3-ICG ([Mn]: 2 mg/kg, four dose), 2 × MnCO3-ICG ([Mn]: 4 mg/kg, four dose), 4 × MnCO3-ICG ([Mn]: 8 mg/kg, four dose), MnCO3-ICG ([Mn]: 2 mg/kg + L (0.5 W/cm2), one dose) and MnCO3-ICG ([Mn]: 2 mg/kg + L (0.5 W/cm2), three dose), respectively. **Figure S25**. Body weight curves of BALB/C tumor-bearing mice after various treatments (n = 5). Groups 1, 2, 3, 4, 5 and 6 were used to represent PBS, MnCO_3_-ICG ([Mn]: 2 mg/kg, four dose), 2 × MnCO_3_-ICG ([Mn]: 4 mg/kg, four dose), 4 × MnCO_3_-ICG ([Mn]: 8 mg/kg, four dose), MnCO_3_-ICG ([Mn]: 2 mg/kg + L (0.5 W/cm^2^), one dose) and MnCO_3_-ICG ([Mn]: 2 mg/kg + L (0.5 W/cm^2^), three dose), respectively. **Figure S26**. Hematoxylin & eosin (H&E)-stained of the organ harvested from mice in different formulations after the 14-day treatment period. Groups 1, 2, 3, 4, 5 and 6 were used to represent PBS, MnCO_3_-ICG ([Mn]: 2 mg/kg, four dose), 2 × MnCO_3_-ICG ([Mn]: 4 mg/kg, four dose), 4 × MnCO_3_-ICG ([Mn]: 8 mg/kg, four dose), MnCO_3_-ICG ([Mn]: 2 mg/kg + L (0.5 W/cm^2^), one dose) and MnCO_3_-ICG ([Mn]: 2 mg/kg + L (0.5 W/cm^2^), three dose), respectively, (scale bar, 100 μm). **Figure S27**. (a) Intracellular ·OH generation after incubation with MnCO_3_-ICG in different buffer solutions (pH 7.4, pH 6.5, and pH 6.5 with laser) detected by DCFH-DA probe. The blue and green fluorescence indicate cell nucleus and DCFH-DA, respectively (scale bar, 50 μm). (b) The fluorescence intensity of ·OH,based on DCFH-DA staining resuts in panel (a) (**P < 0.05). **Figure S28**. (a) CLSM observation on the intracellular distribution of lipoperoxides in Hep 1–6 cells after incubation with PBS and MnCO_3_-ICG for 24 h. The red fluorescence is the lipid ROS in cells and membranes after the staining with BODIPY-C11 (scale bar, 50 μm). (b) lipoperoxides, based on BODIPY staining results in panel (a) (***P < 0.001). **Figure S29**. (A) CLSM observation on the changes in the mitochondrial membrane potential of Hep 1–6 cells after incubation with different concentration of MnCO_3_-ICG (scale bar, 50 μm). (B) The membrane potential (ΔΨm) changes, assessed by JC-1staining (***P < 0.001). **Figure S30**. LDH release assay of Hep 1–6 cells after incubation with different concentration of MnCO_3_-ICG (***p < 0.001).

## Data Availability

The datasets used and analyzed during the current study are available from the corresponding author on reasonable request.
