# Supplementary material for: Intracellular marriage of bicarbonate and Mn ions as “immune ion reactors” to regulate redox homeostasis and enhanced antitumor immune responses
Source: J Nanobiotechnology. 2022 Apr 19;20:193. doi: 10.1186/s12951-022-01404-x (PMC9020034; doi:10.1186/s12951-022-01404-x)
Supplement: Supplementary file 1 — Additional file 1: Figure S1. Typical TEM images of MnCO3-ICG complexes prepared at different feeding ratios of ICG and MnCl2·4H2O. Figure S2. (a) the absorption spectra of MnCO3-ICG complexes at different time. (b) Typical TEM images of MnCO3-ICG complexes prepared at different time. Figure S3. (a) TEM imaging of MnCO3-ICG-PAH complexes prepared with different feeding ratios of MnCO3-ICG and PAH. (b) Photographs of MnCO3-ICG-PAH complexes after being incubated in H2O, PBS (7.4), cell medium (DMEM), and serum (FBS) for 12 h. (c) The hydrodynamic diameter of MnCO3-ICG-PAH complexes with different feeding ratios. Figure S4. (a) X-ray powder diffraction pattern of the MnCO3-ICG and the re-assembled nanoformulation after incubation in the acidic buffer solutions. (b) XPS spectra for MnCO3-ICG and the re-assembled nanoformulation after incubation in the acidic buffer solutions. XPS spectra of Mn2p for (c) for MnCO3-ICG and (d) the re-assembled nanoformulation after incubation in the acidic buffer solutions. Figure S5. (a) Ultrasound images of the generation of CO2 after CaCO3 incubation with different time (pH 5.8 + H2O2). (b) Ultrasound signal intensity of the generation of CO2 after CaCO3 incubation with different time (pH 5.8 + H2O2). (c) The typical chromatograms of CO2 generated after MnCO3-ICG incubation with acidic environment. Figure S6. (a) Colorimetric analysis of the Fenton-like reaction for MB decolorization with different concentration of HCO3− in acidic environment. (b) Colorimetric analysis of the Fenton-like reaction for MB decolorization with different treatment. (c) Colorimetric analysis of the Fenton-like reaction for MB decolorization after different reaction time with acidic environment (pH 5.8). Figure S7. (a) Colorimetric analysis of the Fenton-like reaction for MB decolorization after pulling a vacuum. (b) Bar plot showing the of MB after different treatments. Figure S8. The absorption spectra of ICG, Mn-ICG and MnCO3-ICG. Figure S9. (a) Hep G2 ce [file 12951_2022_1404_MOESM1_ESM.docx]

**Electronic Supplementary Material:**

**Intracellular marriage of bicarbonate and Mn ions as “immune ion reactors” to regulate redox homeostasis and enhanced antitumor immune responses**

Yushuo Feng, Yaqing Liu, Xiaoqian Ma, Lihua Xu, Dandan Ding, Lei Chen, Zongzhang Wang, Ruixue Qin, Wenjing Sun, Hongmin Chen*

State Key Laboratory of Molecular Vaccinology and Molecular Diagnostics and Center for Molecular Imaging and Translational Medicine, School of Public Health, Xiamen University, Xiamen 361102, China

E-mail: hchen@xmu.edu.cn

**Supplemental Experiments**

**Materials**

Poly (allylamine hydrochloride) (PAH), hydrogen peroxide (H_2_O_2_), methylene blue (MB), 4-nitrophenyl chloroformate 3-(4,5-dimethylthiazol-2-yl)-2,5diphenyltetrazolium bromide (MTT) and AM/PI dual-staining kit were purchased from Sigma-Aldrich. Indocyanine green (ICG) was purchased from ACROS ORGANICS. Manganese chloride tetrahydrate (MnCl_2_·4H_2_O) and ammonia bicarbonate (NH_4_HCO_3_) were purchased from Sinopharm Chemical Reagent CO, Ltd, China. Malondialdehyde (MDA) assay kit was purchased from Elabscience Biotechnology Co. Ltd. 2',7'-Dichlorodihydrofluorescein diacetate (DCFH-DA), lactate dehydrogenase (LDH) Assay Kit and Lyso-Tracker Red were purchased from Shanghai Beyotime Biotechnology Co., Ltd. JC-1 were purchased from MedChemExpress. BODIPY™ 581/591 C11 (Lipid Peroxidation Sensor) were purchased from thermo fisher scientific.

**Characterization**

Transmission electron microscopy (TEM) was carried out on a JEOL 1200EX transmission electron microscope. Absorption spectra were recorded using a UV–vis spectrometer (Agilent Cary60, USA). Particle size and surface charge were measured by Zetasizer Nano-ZS size analyzer (Malvern Instruments, Malvern, UK). The crystal structure was analyzed by a PANalytical X’Pert PRO powder X-ray diffractometer (XRD) with Cu Kα1 radiation. The fluorescence images of cells were taken on a laser scanning confocal microscopy (Olympus FV1200, Japan). Photothermal irradiation was carried out using a semiconductor laser unit (KS3-11312-110, BWT, Beijing Kaipulin). MR imaging was conducted on a 9.4 T small animal MRI system (Bruker, Germany). Fluorescence imaging was conducted on IVIS Lumina II system.

**Degradation and drug release studies**

MnCO_3_-ICG was incubated with PBS solutions (pH = 5.8 and 7.4) for different durations. At the given time points, the solutions were centrifuged to collect the precipitation and supernatant. The released of Mn^2+^ was separately measured by *T_1_*-MRI (9.4 T). The precipitation was also measured by the TEM to prove the degradation behavior of MnCO_3_. The generation of CO_2_ bubbles with MnCO_3_-ICG ([Mn]: 50 μg/mL) for different time were detected by an ultrasound imaging system with the acidic environment (pH 5.8 + H_2_O_2_).

**Measurement of photothermal performance**

MnCO_3_-ICG complexes with different concentrations ([ICG]: 18.2, 36.4 and 72.8 μg/mL) were suspended in tubes and irradiated by a NIR laser at 808 nm (0.5 W/cm^2^, 5 min). The temperature and images of solution were recorded every 1 min by an Infrared radiation (IR) camera.

**Cell culture**

4T1 (mouse breast cancer) cell lines, Hep G2 (Human Hepatocellular Carcinoma Cell) cell lines, U87MG (human glioblastoma) cell lines, 3T3 cell lines (mouse embryo fibroblasts), and L02 (human hepatocyte) cell lines were cultured at 37 ^o^C within 5% CO_2_.

**The uptake of MnCO_3_-ICG under the multicellular spheroids (MCSs)**

4T1 cells (3000 cells per well) were seeded into 96-wells plate containing 1% agarose to form MCSs. In this experiment, the MnCO_3_-ICG ([Mn]: 10 μg/mL) were added to the dishes of the MCSs for 24 h. The uptake after co-incubating with complexes were captured by a laser scanning confocal fluorescence microscope.

**Oxidative stress assessment**

For ROS observation, 4T1 cells were seeded in observation dish at a density 10^5^ cells for 24 h. Then, 4T1 cells were treated with MnCO_3_-ICG and Mn-ICG at the different Mn concentration (5, 10, 20 μg/mL) for 24 h. After being washed with PBS, the cells were incubated with hoechst33342 (5 μg/mL) and DCFH-DA (5 μM) for 30 min. Subsequently, the fluorescence images were acquired by a laser scanning confocal fluorescence microscope.

For quantitative analysis the generation of ROS, 4T1 cells were seeded in six-well plates at a density 10^5^ cells for 24 h. After that, 4T1 cells were treated with MnCO_3_-ICG at the different Mn concentration (5, 10, 20 μg/mL) for 24 h. After washing with PBS for three times, the cells were stained with DCFH-DA and analyzed by flow cytometer (ACEA Biosciences).

To evaluate the intracellular lipid peroxidation levels, Malondialdehyde (MDA) assay kit and BODIPY C11 were selected as the testing kits. 4T1 cells were respectively seeded in six-well plates and observation dish at a density 10^5^ cells for 24 h. Then, the cells were treated with MnCO_3_-ICG ([Mn]: 5 μg/mL) and Mn-ICG ([Mn]: 5 μg/mL) for 24 h. After the incubation, 4T1 cells in observation dish were washed with PBS for three times and stained with hoechst33342 (5 μg/mL) and BODIPY C11 (5 μM), and then obtained the fluorescence images, and 4T1 cells in six-well plates were harvested measured using an MDA Assay Kit by following the vendor’s protocol.

For lactate dehydrogenase (LDH) release assay, 4T1 cells were seeded in 96-well plates (10^4^ cells per well) and incubation for 24 h prior to the experiment. The cells were treated with MnCO_3_-ICG ([Mn]: 5, 10 and 20 μg/mL) and Mn-ICG ([Mn]: 5, 10 and 20 μg/mL) for 24 h. The LDH release was tested according with the vendor’s protocol. Briefly, the cell-only group were were mixed with LDH releasing agent to acquire the maximum LDH release. Then, the supernatant (120 μL) in DMEM-only group, cell-only group, the maximum LDH release group and other experiment group was collected and tested.

To monitor the changes of mitochondrial membrane potential (MMP), JC-1 (a fluorescent lipophilic carbocyanine dye) were selected as detection probe. 4T1 cells were seeded in observation dish at a density 10^5^ cells for 24 h. The cells were treated with MnCO_3_-ICG ([Mn]: 5, 10 and 20 μg/mL) and Mn-ICG ([Mn]: 5, 10 and 20 μg/mL). After incubation for 24 h, the cells were washed with PBS for three times and stained with hoechst33342 (5 μg/mL) and JC-1 (2.5 μM). After washing with PBS for three times, the fluorescence images were acquired on an Olympus FV1200 laser scanning confocal microscope.

For quantitative analysis the changes of MMP, 4T1 cells were seeded in six-well plates at a density 10^5^ cells for 24 h. After that, 4T1 cells were treated with MnCO_3_-ICG ([Mn]: 5, 10 and 20 μg/mL) for 24 h. After washing with PBS for three times, the cells were stained with JC-1 and analyzed by flow cytometer (ACEA Biosciences).

**Animal experiments**

Our animal experiments were conducted on female BALB/c mice, which were purchased from Shanghai SLAC Laboratory Animal Co. Ltd (Shanghai, China). All the experiments were under the guidelines of the Regional Ethics Committee for Animal Experiments and the Care Regulations approved by the Institutional Animal Care and Use Committee of Xiamen University. 4T1 tumor-bearing mice model were established by subcutaneously injecting of 4T1 cells (2*10^6^) suspended 100 μL into the back of the hind leg.

**Biodistribution and blood circulation**

4T1 tumor-bearing female mice were intravenously injected with MnCO_3_-ICG ([Mn]: 2 mg/kg) when the tumor reached 80~100 mm^3^. Then the blood samples were collected from the orbital venous plexus at different time points (0.5, 1, 2, 4, 8, 12, 24 and 48 h). For biodistribution, the major organ and tumor were collected at the same time points. The blood samples and major organ were weighted and dissolved in HNO_3_-H_2_O_2_ solutions. The Mn content were tested by inductively couple plasma mass spectrometry (ICP-MS).

**In vivo imaging**

4T1 tumor bearing female mice were intravenously injected with MnCO_3_-ICG ([Mn]: 2 mg/kg). MR imaging (T1-weighted) was conducted on a 9.4 T small animal MRI system (Bruker, Germany) at 2, 4, 8 and 24 h post-injection time points. Fluorescence imaging was conducted at 2, 4, 8 and 24 h post-injection time points using an IVIS Lumina II in vivo imaging system. At the 4 and 24 h time point, mice were euthanized and dissected. Tumors and major organs were collected for ex fluorescence signals.

**The antitumor efficiency for hepatocellular carcinoma (Hep1-6) cells and Orthotopic Hepatic Tumors**

Female C57BL/6 (18−20 g) purchased from the Animal Care and Use Committee of Xiamen University, were used for assessing the treatment effect. Briefly, mice were anesthetized with 5% chloral hydrate, and then 20 µL Hep 1-6/Luc cells (5×10^6^ per mouse) were injected into the right liver lobe by a laparotomy. About 14 days later, bioluminescence images performed with IVIS Lumina II after intraperitoneal injection of fluorescein substrate to screen out tumor-bearing mice for in vivo therapy. The tumor formation rate was 60%.

After confirming tumor establishment, the tumor-bearing mice were divided randomly into 3 groups (n = 5/group): (1) PBS; (2) MnCO_3_-ICG (2 mg/kg, three doses, termed as CDT); (3) MnCO_3_-ICG ([Mn]:2 mg/kg + L (0.5 W/cm^2^), three doses, termed as PTT + CDT). During the entire treatment period, body weights and in vivo bioluminescence images were recorded after the different treatments. All mice were euthanized on day 24 after tumor therapy, then the tumors were taken out and photographed. The excised tumors were collected for standard H&E staining.


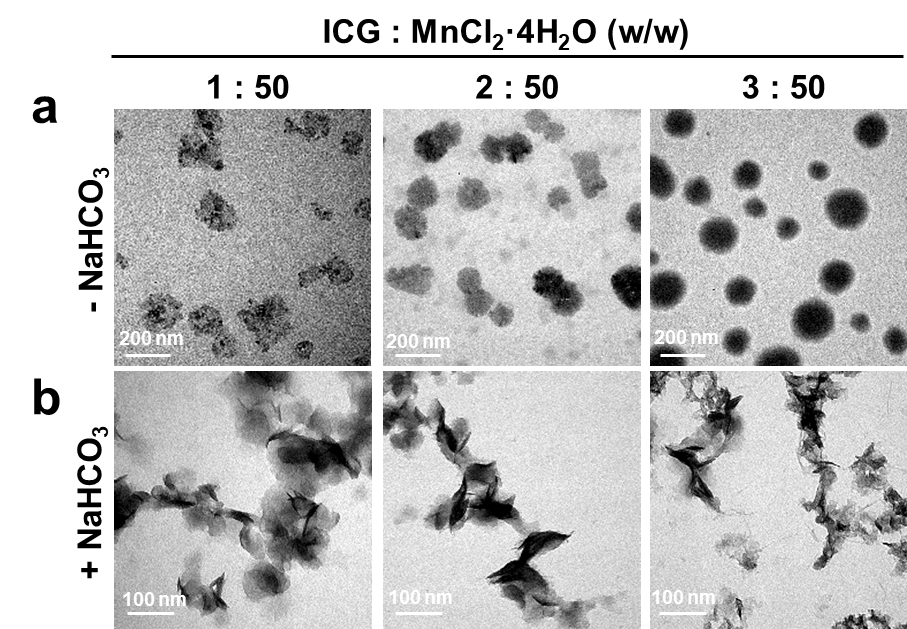


**Figure S1.** Typical TEM images of MnCO_3_-ICG complexes prepared at different feeding ratios of ICG and MnCl_2_·4H_2_O.


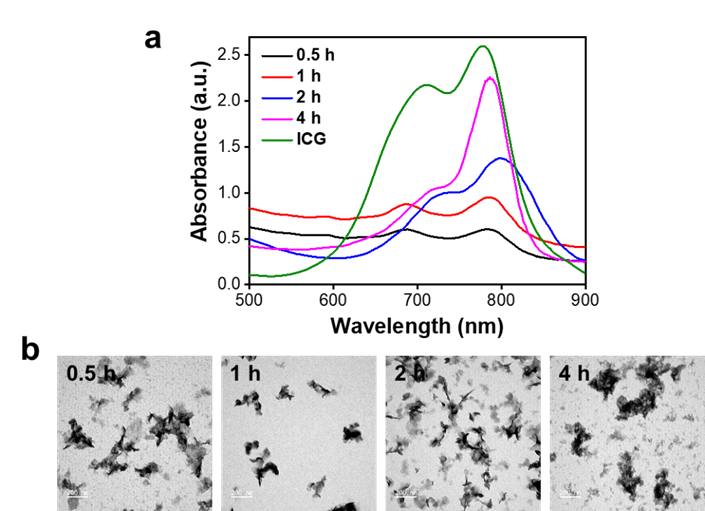


**Figure S2.** (a) the absorption spectra of MnCO_3_-ICG complexes at different time. (b) Typical TEM images of MnCO_3_-ICG complexes prepared at different time.


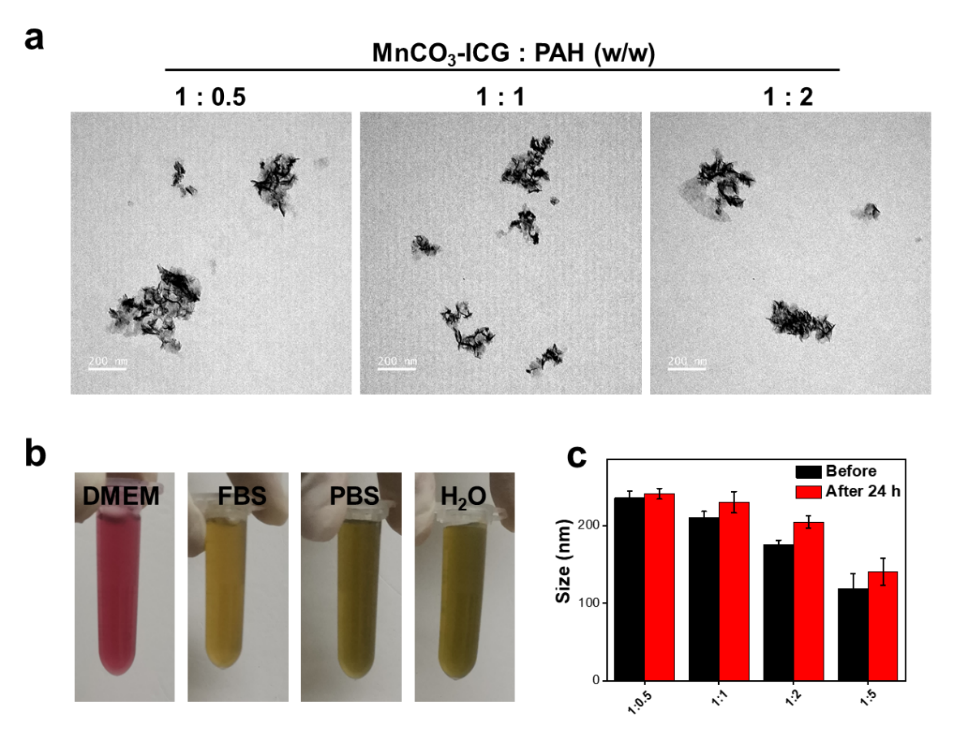


**Figure S3.** (a) TEM imaging of MnCO_3_-ICG-PAH complexes prepared with different feeding ratios of MnCO_3_-ICG and PAH. (b) Photographs of MnCO_3_-ICG-PAH complexes after being incubated in H_2_O, PBS (7.4), cell medium (DMEM), and serum (FBS) for 12 h. (c) The hydrodynamic diameter of MnCO_3_-ICG-PAH complexes with different feeding ratios.


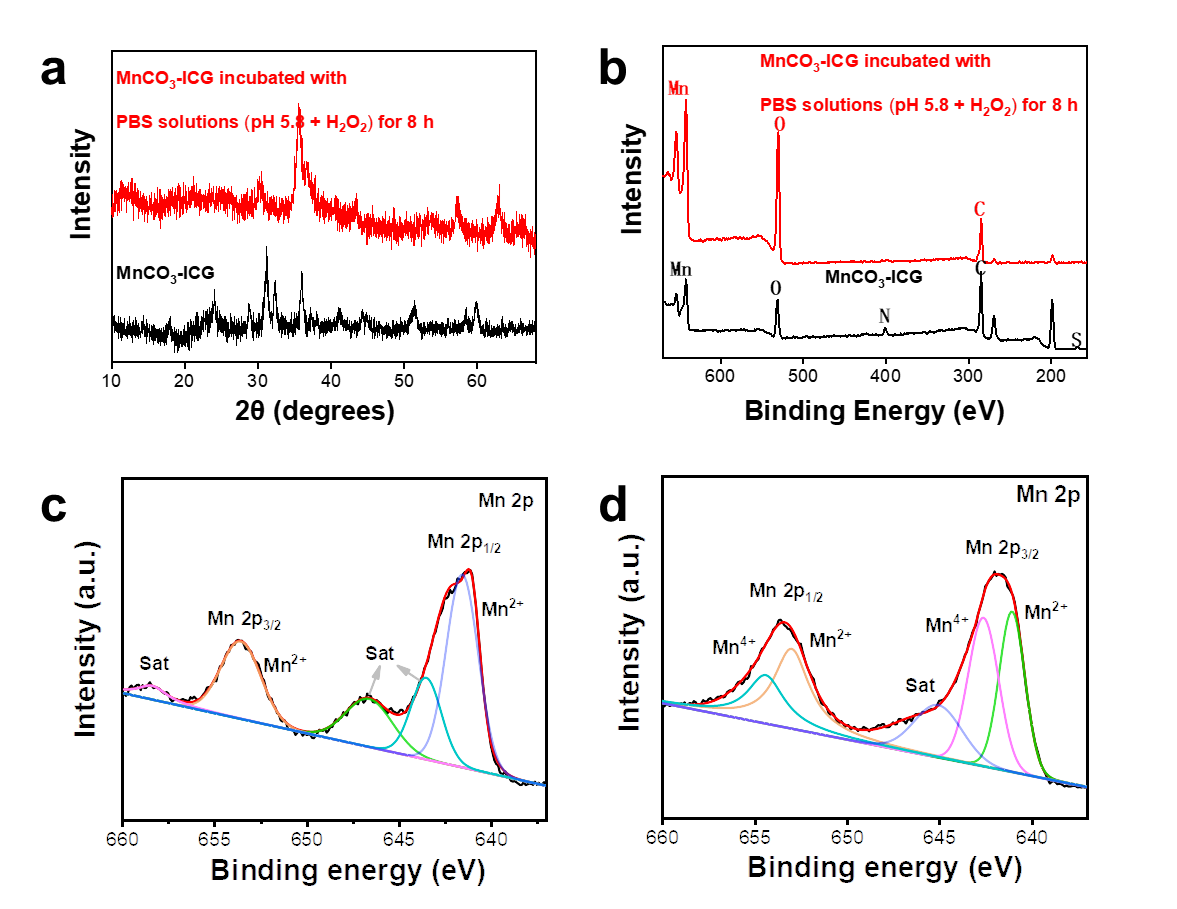


**Figure S4.** (a) X-ray powder diffraction pattern of the MnCO_3_-ICG and the re-assembled nanoformulation after incubation in the acidic buffer solutions. (b) XPS spectra for MnCO3-ICG and the re-assembled nanoformulation after incubation in the acidic buffer solutions. XPS spectra of Mn2p for (c) for MnCO_3_-ICG and (d) the re-assembled nanoformulation after incubation in the acidic buffer solutions.

**
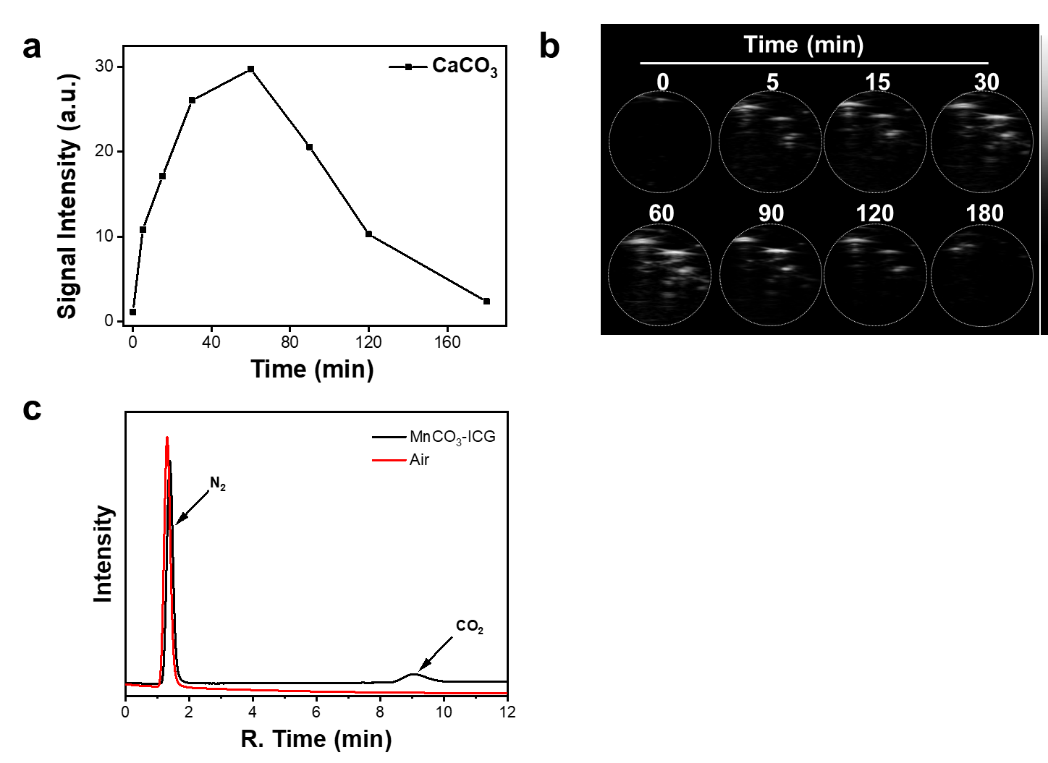
**

**Figure S5.** (a) Ultrasound images of the generation of CO_2_ after CaCO_3_ incubation with different time (pH 5.8 + H_2_O_2_). (b) Ultrasound signal intensity of the generation of CO_2_ after CaCO_3_ incubation with different time (pH 5.8 + H_2_O_2_). (c) The typical chromatograms of CO_2_ generated after MnCO_3_-ICG incubation with acidic environment.


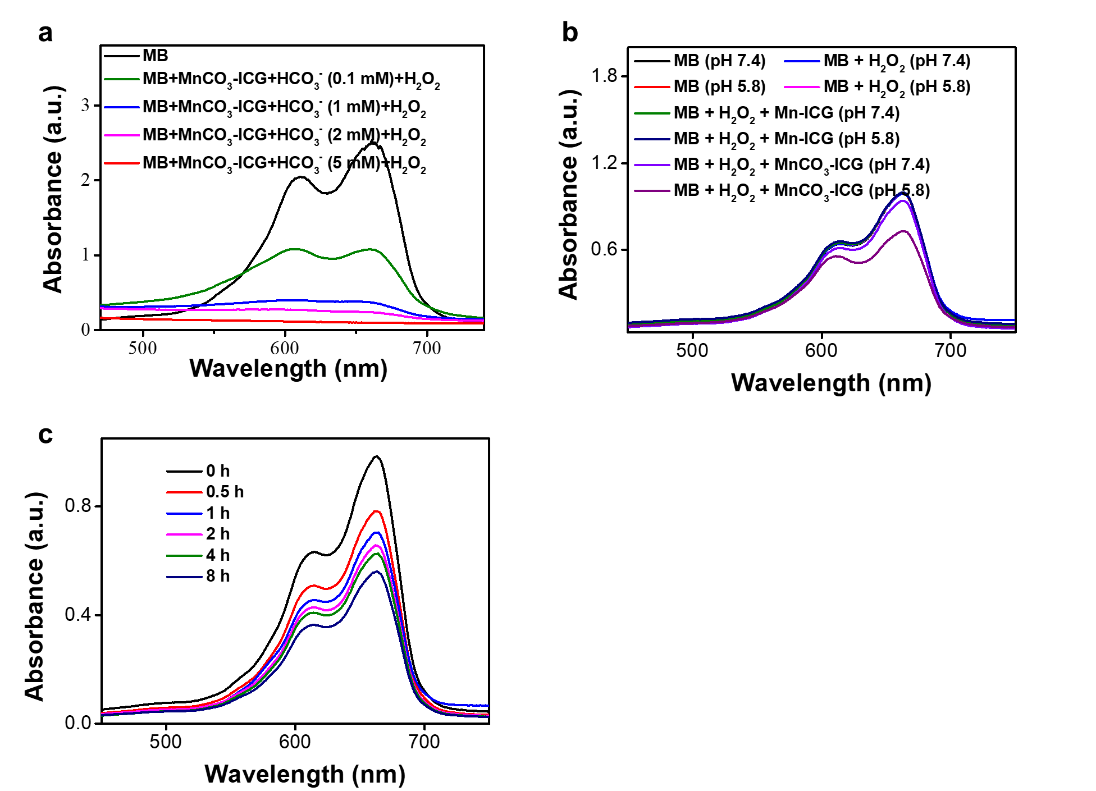


**Figure S6**. (a) Colorimetric analysis of the Fenton-like reaction for MB decolorization with different concentration of HCO_3_^-^ in acidic environment. (b) Colorimetric analysis of the Fenton-like reaction for MB decolorization with different treatment. (c) Colorimetric analysis of the Fenton-like reaction for MB decolorization after different reaction time with acidic environment (pH 5.8).


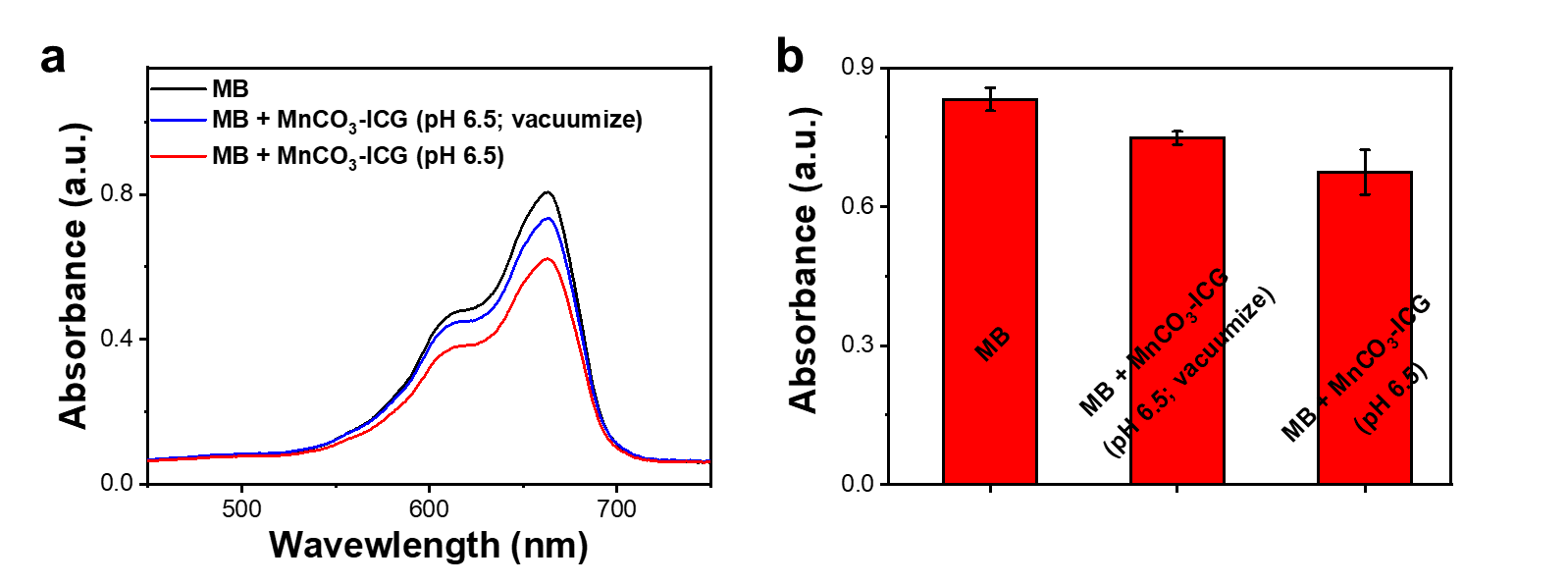


**Figure S7.** (a) Colorimetric analysis of the Fenton-like reaction for MB decolorization after pulling a vacuum. (b) Bar plot showing the of MB after different treatments.

**Figure S8.** The absorption spectra of ICG, Mn-ICG and MnCO_3_-ICG.


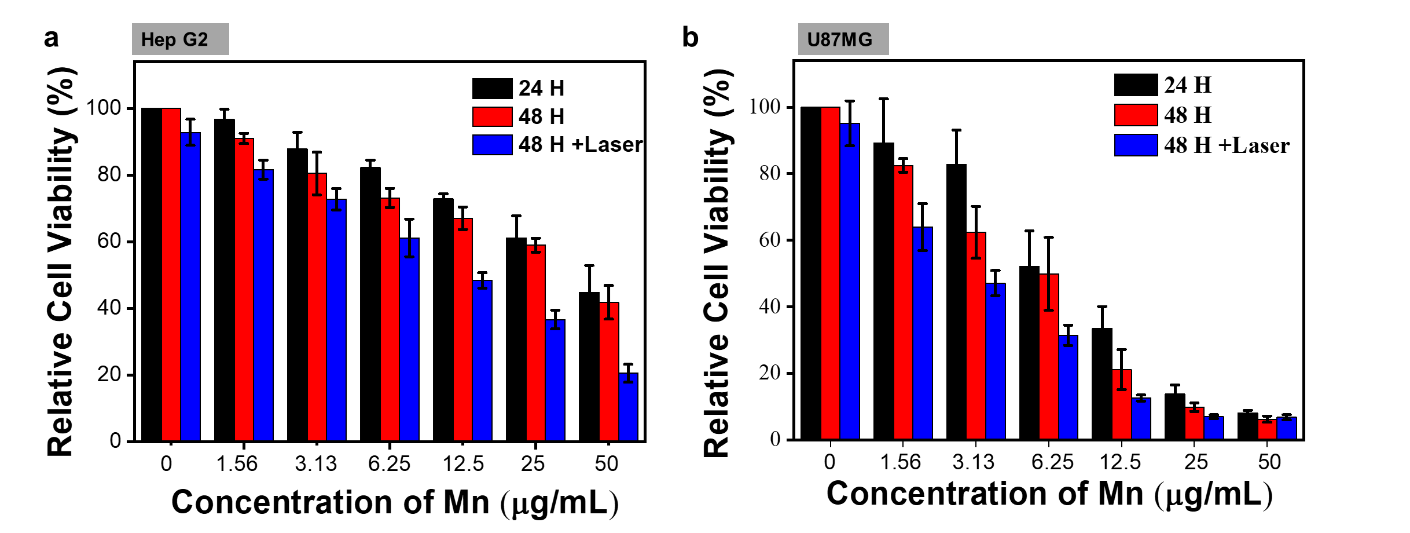


**Figure S9.** (a) Hep G2 cells and (b) U87MG cells incubated with different concentrations of MnCO_3_-ICG with or without 808 nm laser (0.5 W/cm^2^, 5 min) irradiation.


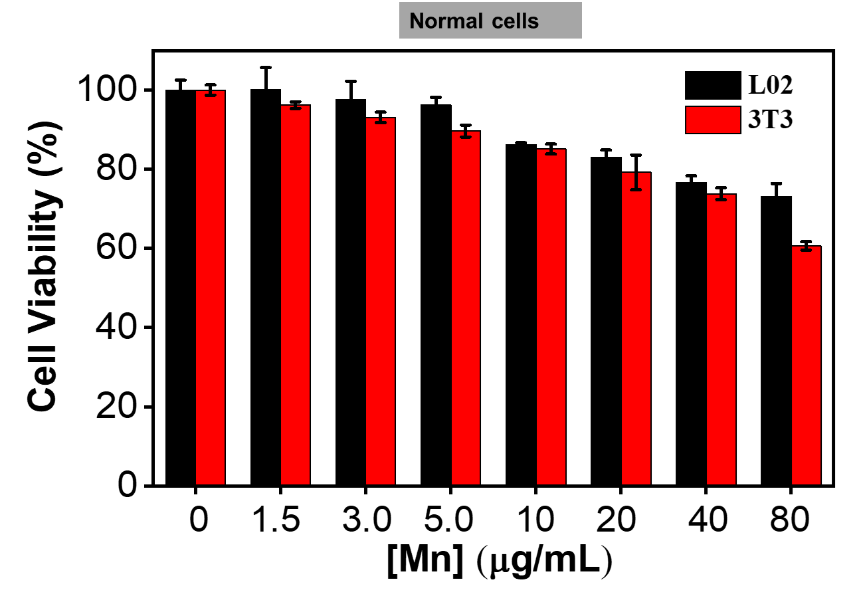


**Figure S10**. Relative cellular viabilities of normal L02 and 3T3 cells incubated with different concentrations of MnCO_3_-ICG.


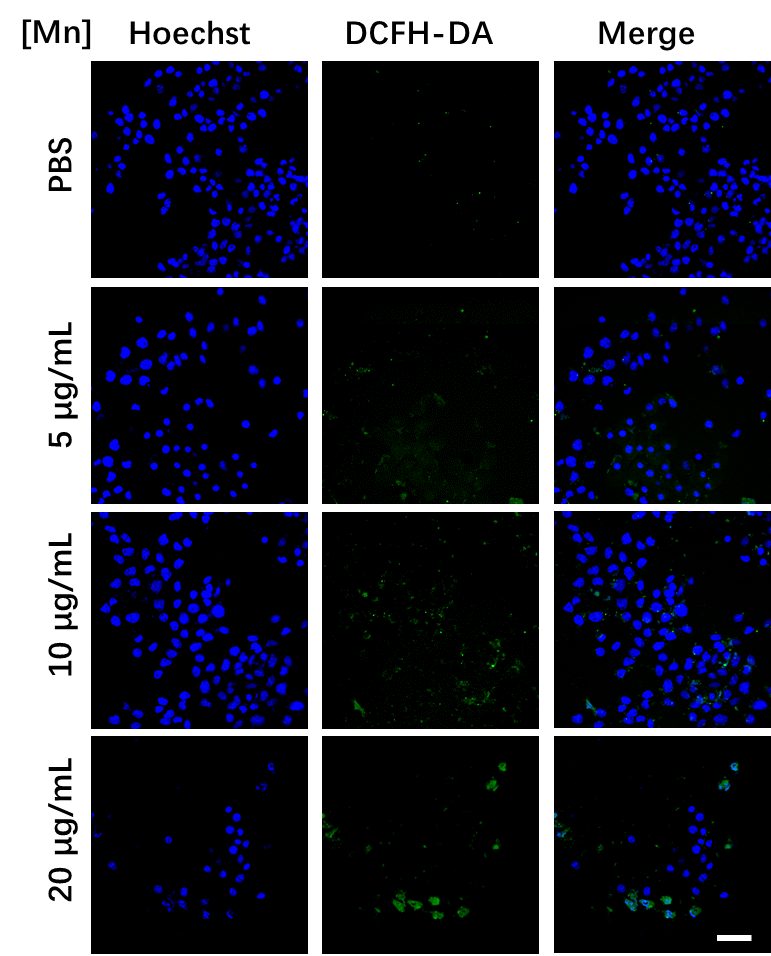


**Figure S11**. Intracellular ·OH generation after incubation with Mn-ICG detected by DCFH-DA probe (scale bar, 50 μm).

**Figure S12**. Lipid damage assessment measured by lipid peroxidation assays (**p* < 0.05).


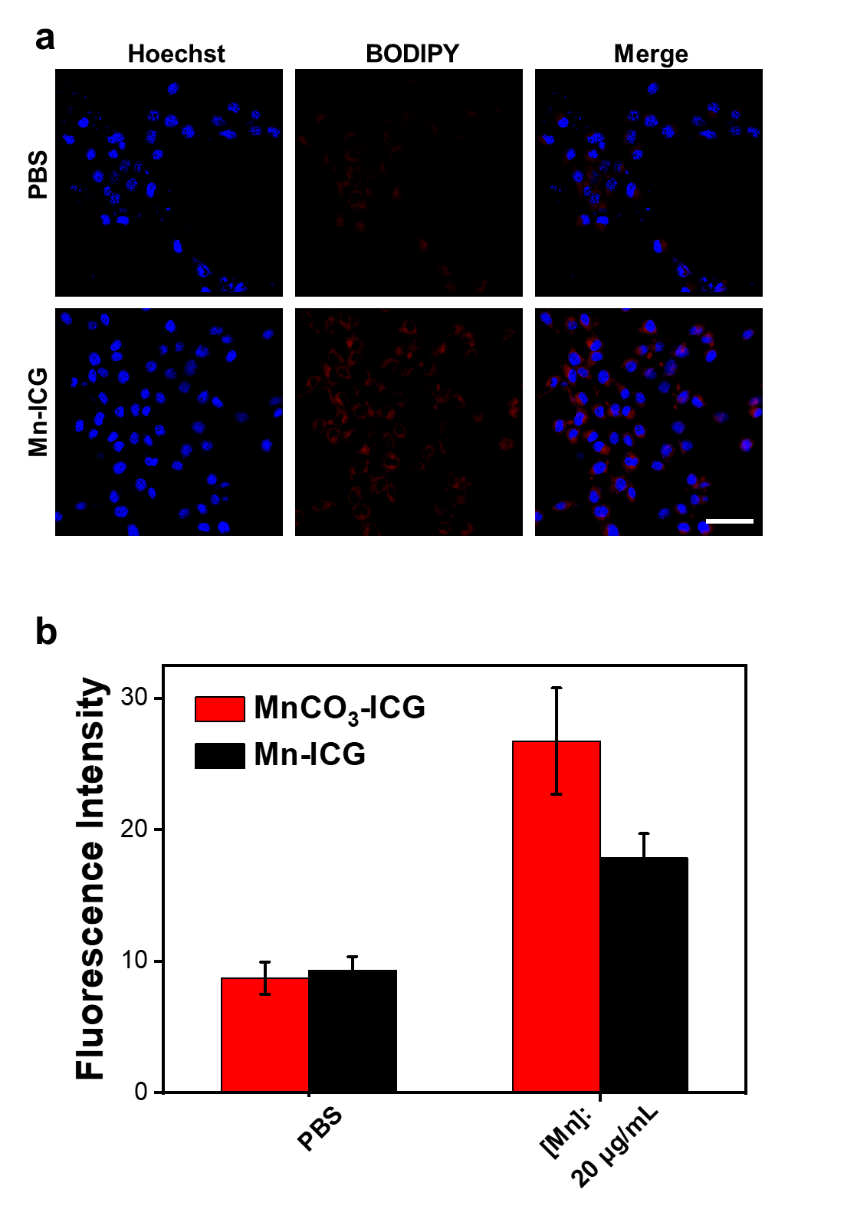


**Figure S13**. (a) CLSM observation on the intracellular distribution of lipoperoxides in 4T1 cells after incubation with PBS and Mn-ICG for 24 hours. The red fluorescence is the lipid ROS ROS in cells and membranes after the staining with BODIPY-C11 (scale bar, 50 μm). (b) lipoperoxides, based on BODIPY staining resuts in panel (a)


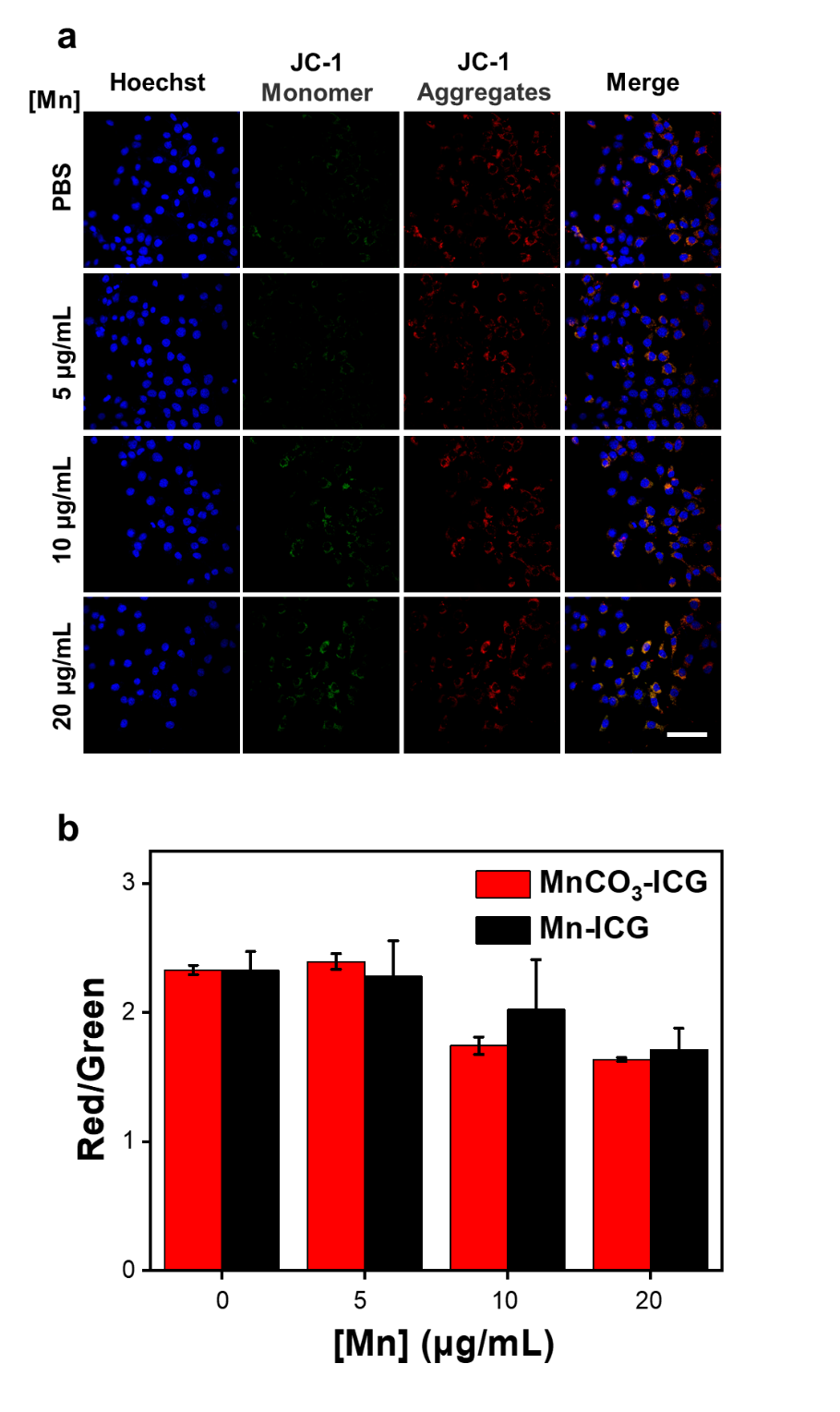


**Figure S14**. (a) CLSM observation on the changes in the mitochondrial membrane potential of 4T1 cells after incubation with different concentration of Mn-ICG (scale bar, 50 μm). (b) The membrane potential (ΔΨm) changes, assessed by JC-1staining.

**Figure S15**. LDH release assay after incubation with different concentration of Mn-ICG.


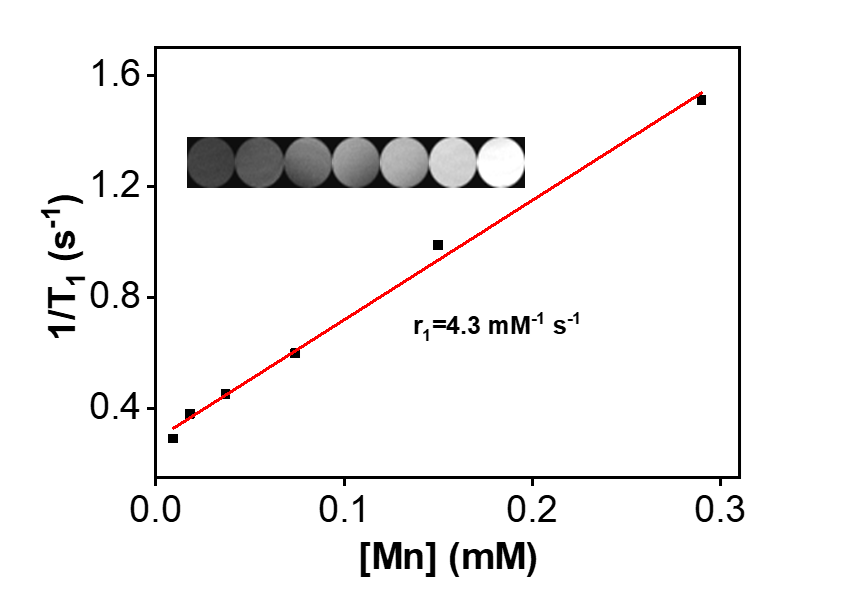


**Figure S16**. T_1_-relaxation rate (r_1_) and T_1_-weighted MR images of MnCO_3_-ICG.


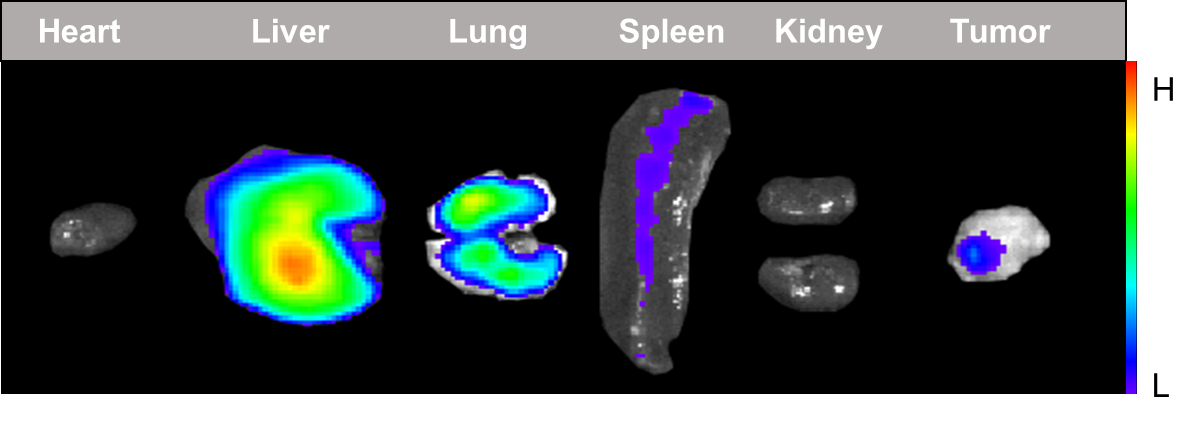


**Figure S17.** Ex vivo fluorescence images of the organs harvested in BALB/C tumor-bearing mice at 24 h post-injection.


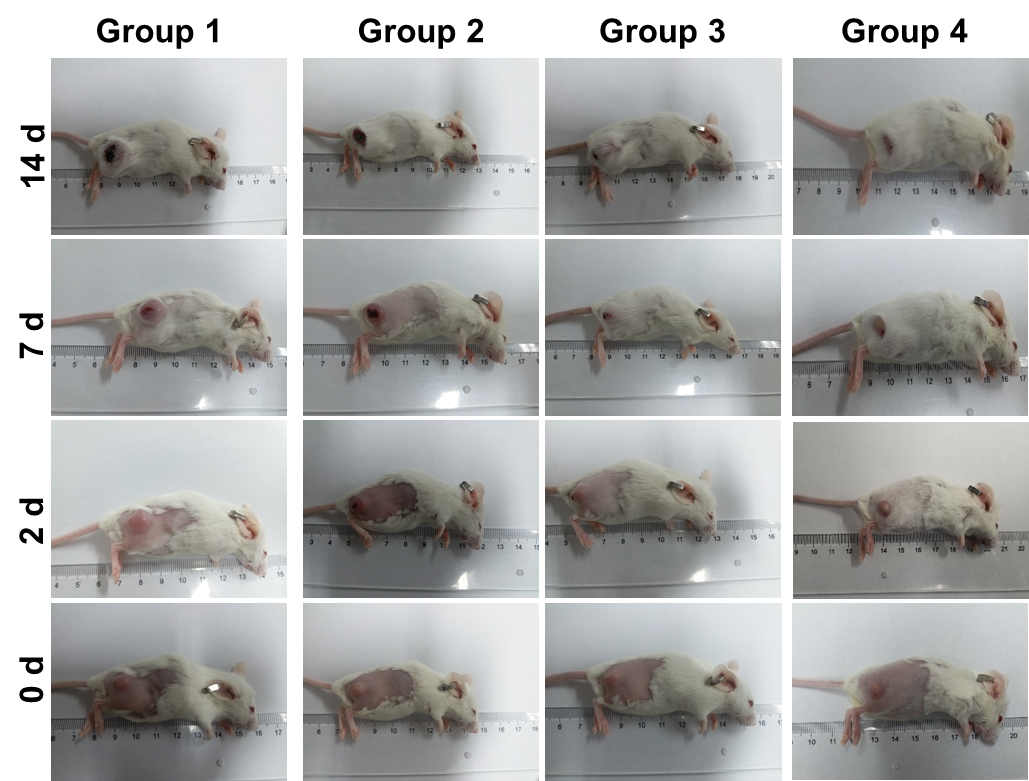


**Figure S18.** Body photos in different formulations after the 14-day treatment period. Groups 1, 2, 3 and 4 were used to represent PBS, MnCO_3_-ICG ([Mn]: 2 mg/kg, four dose), 2×MnCO_3_-ICG ([Mn]: 4 mg/kg, four dose), 4×MnCO_3_-ICG ([Mn]: 8 mg/kg, four dose), respectively.


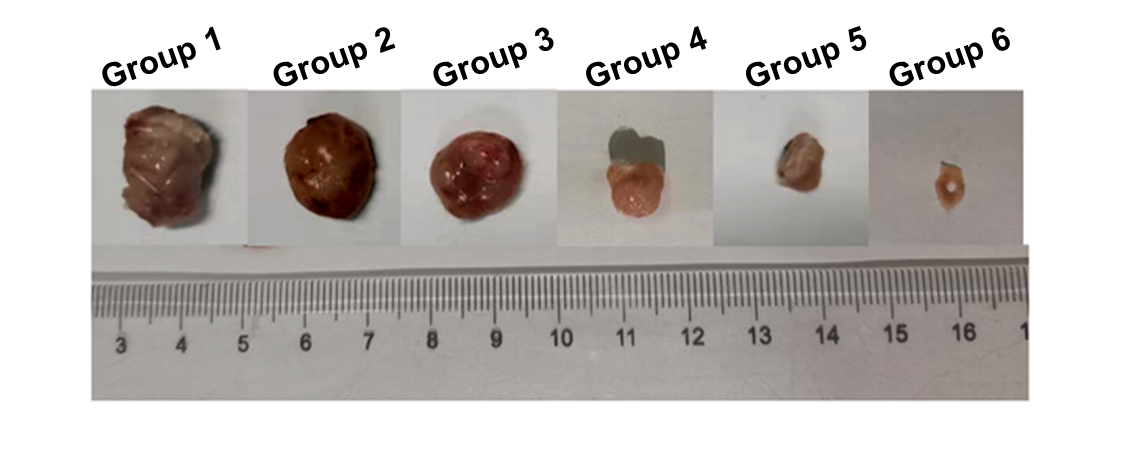


**Figure S19.** Images of representative tumors taken from mice in different formulations after the 14-day treatment period. Groups 1, 2, 3, 4, 5 and 6 were used to represent PBS, MnCO_3_-ICG ([Mn]: 2 mg/kg, four dose), 2×MnCO_3_-ICG ([Mn]: 4 mg/kg, four dose), 4×MnCO_3_-ICG ([Mn]: 8 mg/kg, four dose), MnCO_3_-ICG ([Mn]: 2 mg/kg + L (0.5 W/cm^2^), one dose) and MnCO_3_-ICG ([Mn]: 2 mg/kg + L (0.5 W/cm^2^), three dose), respective.

**Figure S20**. The tumor growth inhibition curves of BALB/C tumor-bearing mice exposed to different formulations after the treatment period. Groups 1, 2, 3, 4, 5 and 6 were used to represent PBS, MnCO_3_-ICG ([Mn]: 2 mg/kg, four dose), 2×MnCO_3_-ICG ([Mn]: 4 mg/kg, four dose), 4×MnCO_3_-ICG ([Mn]: 8 mg/kg, four dose), MnCO_3_-ICG ([Mn]: 2 mg/kg + L (0.5 W/cm^2^), one dose) and MnCO_3_-ICG ([Mn]: 2 mg/kg + L (0.5 W/cm^2^), three dose), respective.


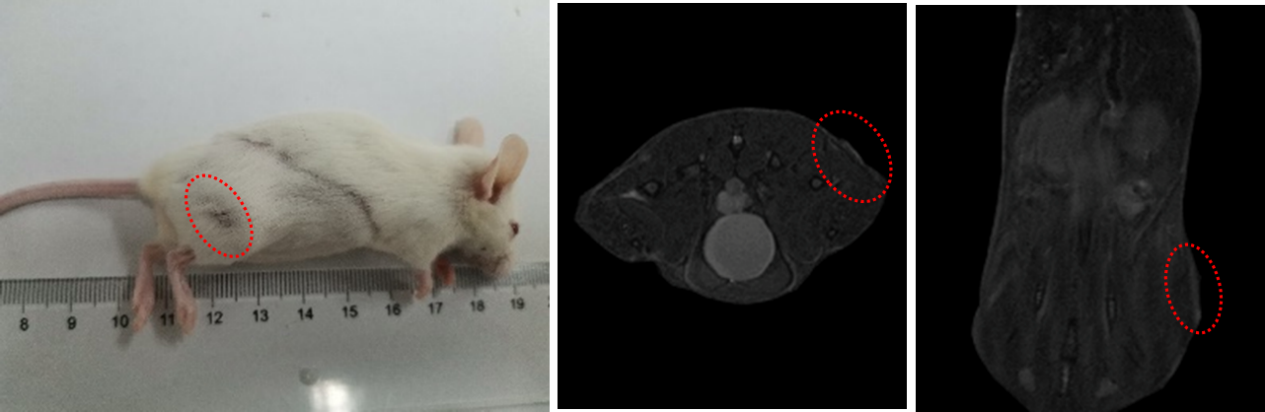


**Figure S21**. The T2-MR imaging and digital photos of mice BALB/C tumor-bearing after the 21-day treatment period (group 6). Groups 6 was used to represent MnCO_3_-ICG([Mn]: 2 mg/kg + L (0.5 W/cm^2^), three dose), respectively.


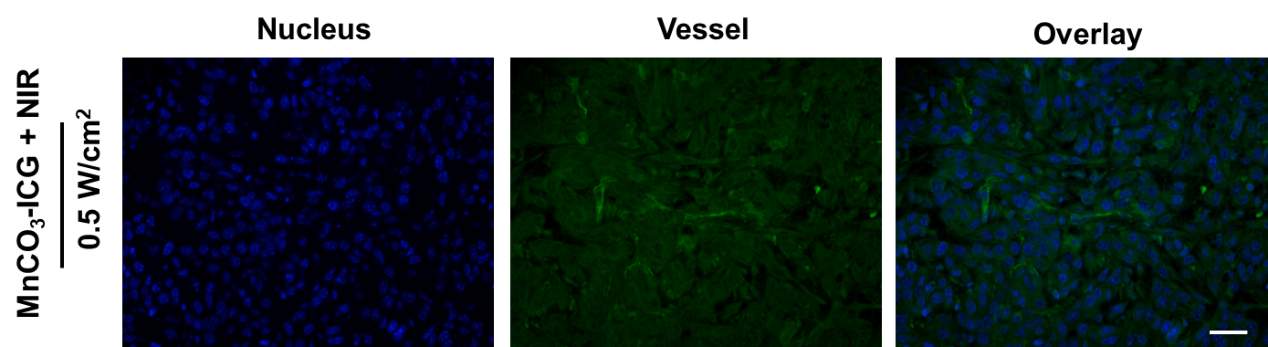


**Figure S22.** Representative immunofluorescence staining of nucleus (blue) and vessel (green) on the tumor slices collected 14-day after laser irradiation (scale bar, 50 μm).


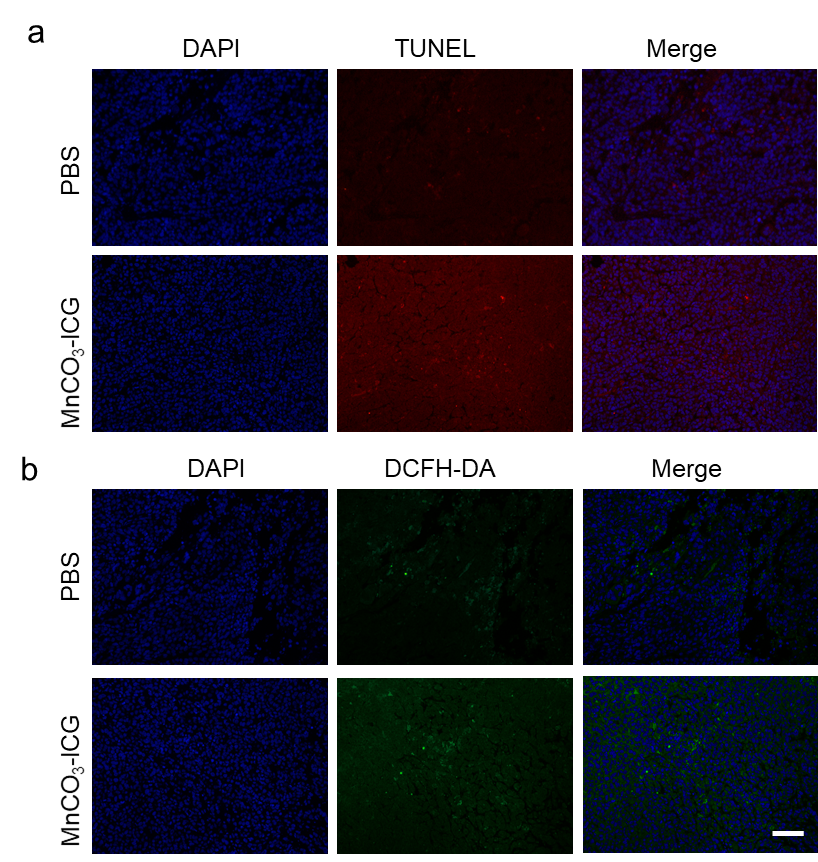


**Figure S23.** Representative immunofluorescence staining of TUNEL (nucleus (blue) and apoptotic cells (red)) and ROS (nucleus (blue) and ROS (green)) on the tumor slices. (scale bar, 200 μm)


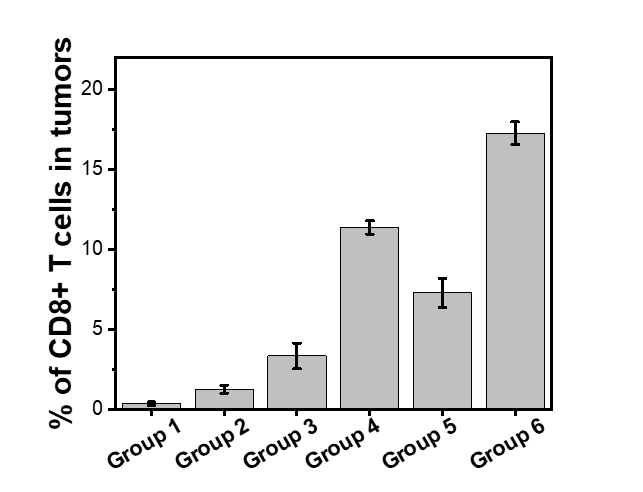


**Figure S24**. The flow cytometric histograms showing the intratumor infiltration of the effector T cells (CD8+ T cells). Groups 1, 2, 3, 4, 5 and 6 were used to represent PBS, MnCO_3_-ICG ([Mn]: 2 mg/kg, four dose), 2×MnCO_3_-ICG ([Mn] : 4 mg/kg, four dose), 4×MnCO_3_-ICG ([Mn] : 8 mg/kg, four dose), MnCO_3_-ICG ([Mn] : 2 mg/kg + L (0.5 W/cm^2^), one dose) and MnCO_3_-ICG ([Mn] : 2 mg/kg + L (0.5 W/cm^2^), three dose), respectively.

**Figure S25**. Body weight curves of BALB/C tumor-bearing mice after various treatments (n = 5). Groups 1, 2, 3, 4, 5 and 6 were used to represent PBS, MnCO_3_-ICG ([Mn] : 2 mg/kg, four dose), 2×MnCO_3_-ICG ([Mn] : 4 mg/kg, four dose), 4×MnCO_3_-ICG ([Mn] : 8 mg/kg, four dose), MnCO_3_-ICG ([Mn] : 2 mg/kg + L (0.5 W/cm^2^), one dose) and MnCO_3_-ICG ([Mn] : 2 mg/kg + L (0.5 W/cm^2^), three dose), respectively.


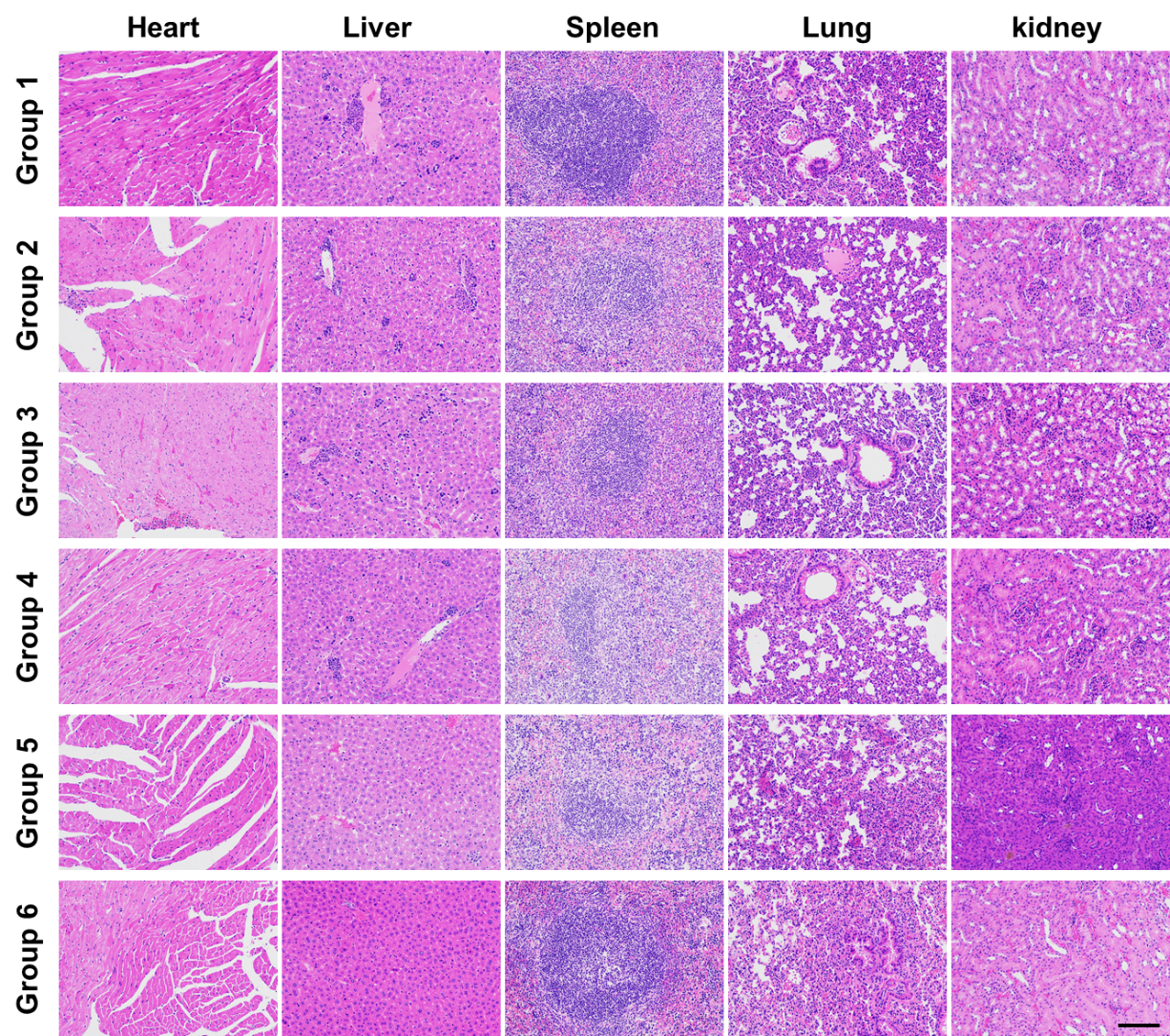


**Figure S26**. Hematoxylin & eosin (H&E)-stained of the organ harvested from mice in different formulations after the 14-day treatment period. Groups 1, 2, 3, 4, 5 and 6 were used to represent PBS, MnCO_3_-ICG ([Mn] : 2 mg/kg, four dose), 2×MnCO_3_-ICG ([Mn] : 4 mg/kg, four dose), 4×MnCO_3_-ICG ([Mn] : 8 mg/kg, four dose), MnCO_3_-ICG ([Mn] : 2 mg/kg + L (0.5 W/cm^2^), one dose) and MnCO_3_-ICG ([Mn] : 2 mg/kg + L (0.5 W/cm^2^), three dose), respectively, (scale bar, 100 μm).


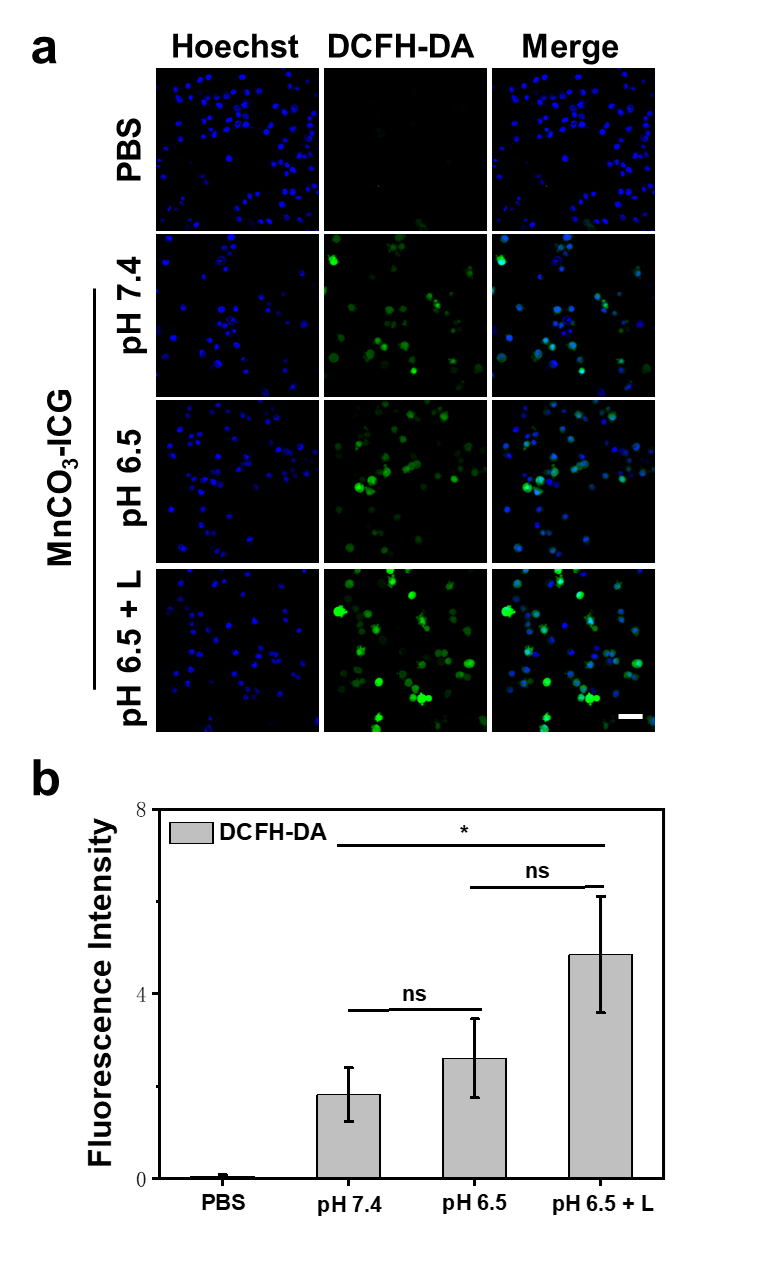


**Figure S27**. (a) Intracellular ·OH generation after incubation with MnCO_3_-ICG in different buffer solutions (pH 7.4, pH 6.5, and pH 6.5 with laser) detected by DCFH-DA probe. The blue and green fluorescence indicate cell nucleus and DCFH-DA, respectively (scale bar, 50 μm). (b) The fluorescence intensity of ·OH ,based on DCFH-DA staining resuts in panel (a) (**P < 0.05).


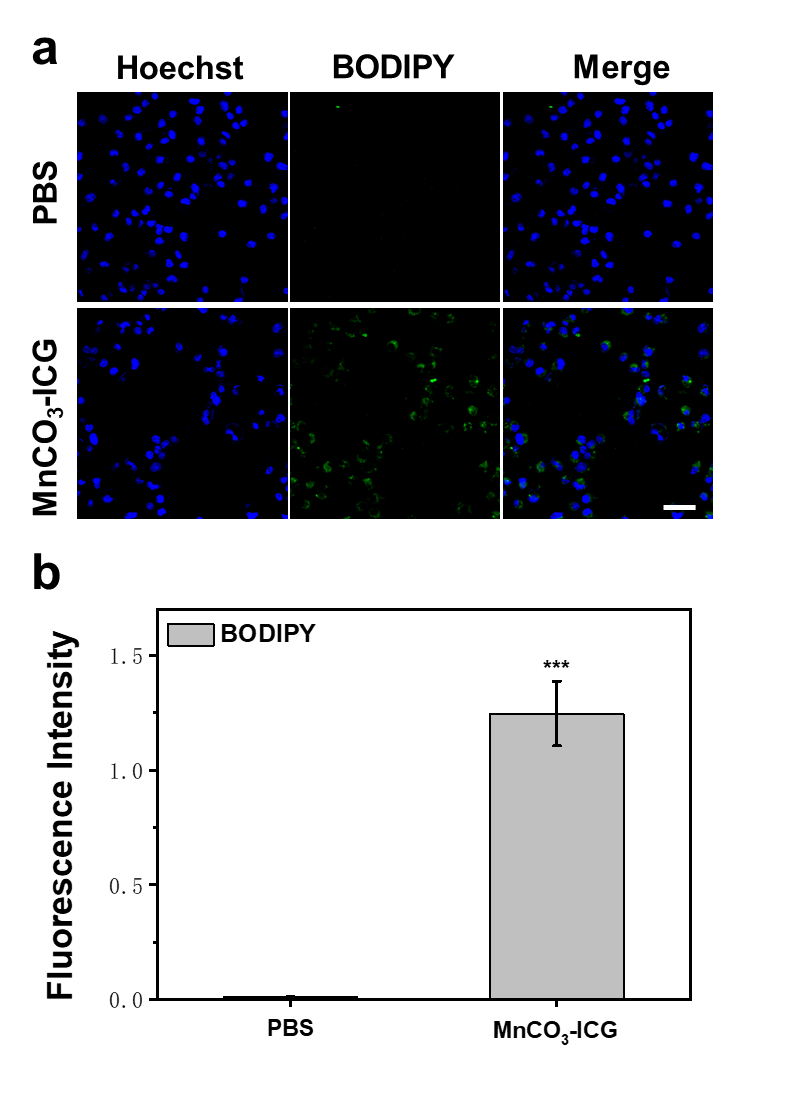


**Figure S28**. (a) CLSM observation on the intracellular distribution of lipoperoxides in Hep 1-6 cells after incubation with PBS and MnCO_3_-ICG for 24 h. The red fluorescence is the lipid ROS in cells and membranes after the staining with BODIPY-C11 (scale bar, 50 μm). (b) lipoperoxides, based on BODIPY staining results in panel (a) (***P < 0.001).


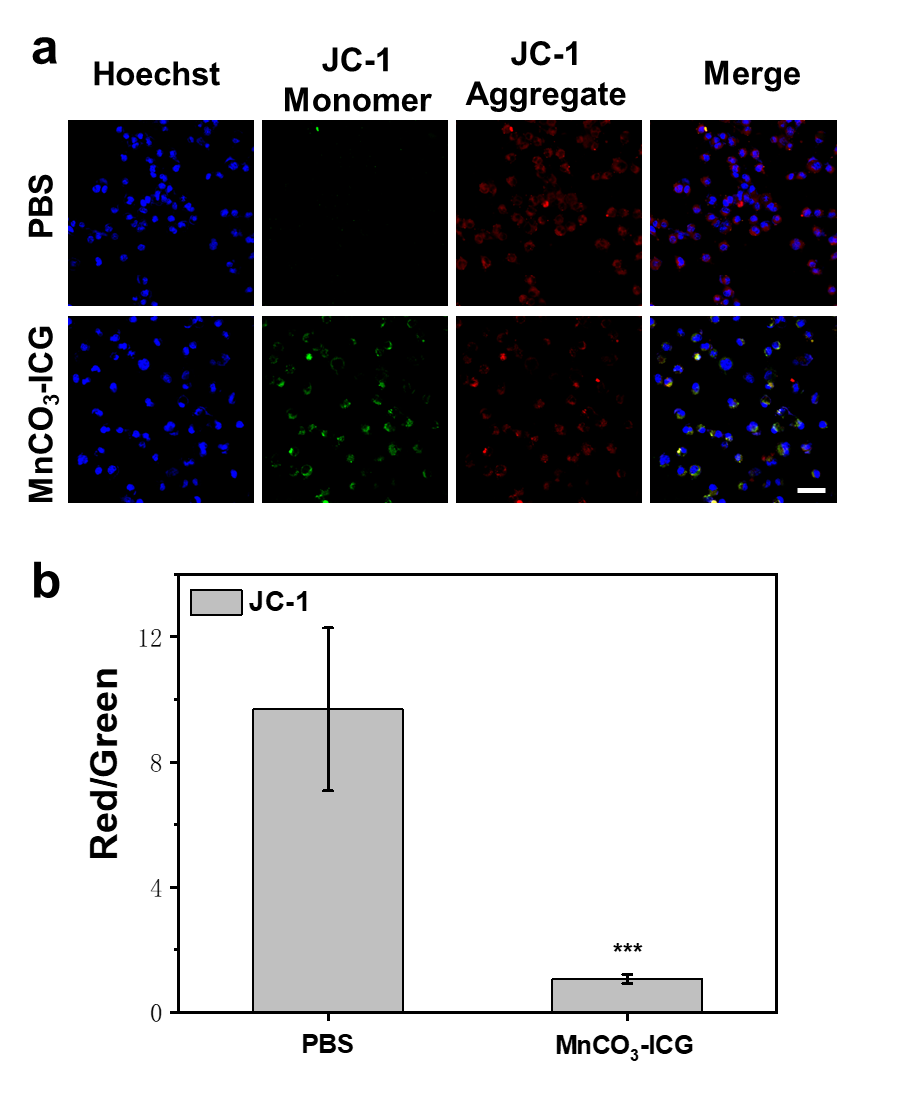


**Figure S29**. (A) CLSM observation on the changes in the mitochondrial membrane potential of Hep 1-6 cells after incubation with different concentration of MnCO_3_-ICG (scale bar, 50 μm). (B) The membrane potential (ΔΨm) changes, assessed by JC-1staining (***P < 0.001).

**Figure S30**. LDH release assay of Hep 1-6 cells after incubation with different concentration of MnCO_3_-ICG (****p* < 0.001).
